# Supplementary material for: Nuclear genetic diversity of head lice sheds light on human dispersal around the world
Source: PLoS One. 2023 Nov 8;18(11):e0293409. doi: 10.1371/journal.pone.0293409 (PMC10631634; doi:10.1371/journal.pone.0293409)
Supplement: S1 File — Including a) Protocol for collecting human lice developed by the Reed lab; b) Questionnaire on inclusivity in global research; and c) Microsatellite data. (PDF) [file pone.0293409.s001.pdf]

Supporting Information for:

"Nuclear genetic diversity of head lice sheds light on human dispersal around the world"

Marina S. Ascunce[1,2,*], Ariel C. Toloza[3], Angélica González-Oliver[4], and David L. Reed[5]
[1]Emerging Pathogens Institute, Department of Plant Pathology, University of Florida, Gainesville, Florida, United States of America
[2]USDA-ARS Center for Medical, Agricultural, and Veterinary Entomology, Gainesville, Florida, United States of America
[3]Centro de Investigaciones de Plagas e Insecticidas (CONICET-UNIDEF), Villa Martelli, Buenos Aires, Argentina
[4]Departamento de Biología Celular, Facultad de Ciencias, Universidad Nacional Autónoma de México, Ciudad de México, México
[5]Florida Museum of Natural History, University of Florida, Gainesville, Florida, United States of America
[*]Corresponding author E-mail: marina.ascunce@usda.gov (MSA)

Nuclear genetic diversity of head lice sheds light on human dispersal around the world", Ascunce et. al. (2023), PONE-D-22-28588

This Supporting information includes:

- Protocol for collecting human lice developed by the Reed lab
- Questionnaire on inclusivity in global research
- Microsatellite data

Protocol for collecting human lice developed by the Reed lab

This protocol was provided to collectors with a kit and a spreadsheet.

Louse Collecting Kit Contents: Please store your collection kit in the Freezer (best) or refrigerator. This will prevent the ethanol in the tubes from evaporating. If the ethanol evaporates, please re-fill with 95% ethanol.

1. Louse comb
   -Run the comb through the hair starting from the very base of the hair – lice stay close to the skin. Collect lice if found using forceps and place them in the labeled vial containing ethanol.

2. Forceps
   -Use forceps to transfer the lice from the comb (or paper) to the ethanol-filled tubes.
   Try not to squish the lice with the forceps when moving them. Alternatively, you can dab the forceps in some ethanol first, and then touch the louse, sometimes the wet surface causes the louse to stick to the forceps.

3. Box with 25 tubes (2 ml) filled with 95% ethanol. The tubes are labeled with an individual identification code
   -Each tube will contain lice from one host individual. Make sure lice are completely submerged in ethanol.

4. Spreadsheet to provide louse information
   -Tubes should be numbered and each number should match the number on the spreadsheet with the information. Please include information such as: locality, if the lice were collected from the head or the clothes, and any other information you feel may be important.

General instructions:

- Lice are dorsoventrally flattened and generally appear brown or red because they feed on blood. Their legs are modified into claws for hanging onto hair.
- Caution for double infestations—head and body lice on the same person. Please if a person present double infestation, collect head lice in one tube and body lice in a separate tube, and make a note that these two vials represent head and body lice from the same person.

Collection Procedure:

1. From each person's head, collect as many lice as you can (at least 10 if possible) and put them in a tube. If body lice are evident, place those in a different tube; if possible collect also a minimum of 10 body lice. Each tube should contain the lice from only one person and should only contain head OR body lice. Make sure lice are completely submerged in the ethanol (otherwise, the lice will rot).

2. Each tube has a unique number that matches the number on the spreadsheet provided. Please fill the excel spreadsheet.
   a. Where the collection took place: country, town, region.
   b. When the lice were collected.
   c. If the lice were collected from the head or the clothing.

3. We have also placed a piece of paper with the same unique number located within the tube of ethanol itself as a precaution in case the number on the outside of the tube becomes obscured. Please leave this paper in the tube.
4. Clean the comb carefully between persons.
5. Please store the lice in the freezer until shipping.
6. Once you have collected the lice please contact us to arrange shipping.

THANK YOU!

Inclusivity in global research

PLOS' policy on inclusivity in global research aims to improve transparency in the reporting of research performed outside of researchers' own country or community and ensures that PLOS publications reporting global research adhere to high standards for research ethics and authorship. Authors of relevant research articles may be asked to complete the questionnaire below, which outlines ethical, cultural, and scientific considerations specific to inclusivity in global research. This questionnaire may be requested when researchers have travelled to a different country to conduct research, if research uses samples collected in another country, research with Indigenous populations or their lands, or if research is on cultural artefacts. Researchers travelling to another country solely to use laboratory equipment will not normally be required to complete the questionnaire. However, the questionnaire can be requested at the journal's discretion for any submission – if you have been requested to complete this questionnaire by the PLOS journal you submitted to, please do so.

Please complete the questionnaire below and include this as a Supporting Information file with your manuscript. Note that if your paper is accepted for publication, this checklist will be published with your article in the supporting information files. Please ensure that you reference the checklist in the main body of your manuscript. We suggest adding a subsection 'Inclusivity in global research' to your Methods section and adding the following sentence: "Additional information regarding the ethical, cultural, and scientific considerations specific to inclusivity in global research is included in the Supporting Information (SX Checklist)"

The questions have been designed to be applicable to a wide range of study types, and there are subsections for both human subjects research and non-human subjects research. If any of the questions are not relevant to your research please mark them as "N/A" as appropriate.

Ethical considerations, permits and authorship

This section is applicable to all research types.

Provide details as to who granted permissions and/or consent for the study to take place in the Methods section of your manuscript. This should include the names of **all** ethics boards, governmental organizations, community leaders or other bodies that provided approval for the study. If individuals provided approval refer to these people by their role or title but do not list their name(s).

Reported on page number: Manuscript PONE-D-22-28588, page 9, Ethic Statement: The Institutional Review Board of the University of Florida exempted the study from review (Exemption of Protocol #2009-U-0422) and waived the need for written informed consent of the participants. This exemption is issued based on the United States Department of Health and Human Services (HHS) regulations. Specifically, HHS regulation 45 CFR part 46 applies to research activities involving human subjects. Because louse removal was voluntary and no information was recorded that would allow the individuals to be identified directly or through identifiers linked to them, the University of Florida waived the need for written informed consent from the participants.

In addition, the guidelines needed for each institution or louse collection center where lice were collected were followed. Since there is a large number of sites and collectors, plus some of the collections were done in the 2000's, we don't have all the list of institutions that provided permits. However, in all cases the collection of lice was voluntary and no personal information was collected. In case of minors, a parent or legal custodian provided consent.

If there were any deviations from the study protocol after approval was obtained please provide details of these changes in the Methods section of your manuscript.

Reported on page number: No

Did this study involve local collaborators that are residents of the country where the research was conducted or members of the community studied? If you do not have any authors from said communities, please provide an explanation for this below.

Yes, in most cases the collection of lice was done by local collaborators. Two of those collectors: Ariel Toloza (from Argentina) and Angelica Gonzalez-Oliver (from Mexico) have been included as co-authors. Another major contributors of louse samples were Ms. Katie Shepherd (Founder & CEO of The Shepherd Institute for Lice Solutions), and Marieta A. H. Braks (Laboratory for Zoonoses and Environmental, National Institute for Public Health and the Environment, Netherlands). Ms. Shepherd has been collaborating with the Reed lab for many years and she is happy with helping with this research. Ms. Braks is interested in insecticide resistance and disease vector in human lice, which are topics that we dind't include in this manuscript. All collectors have been listed in S1 Table.

Everyone listed as an author should meet PLOS' criteria for authorship and all individuals who meet these criteria should be included in the author byline, rather than the acknowledgements. Authorship criteria is based on the International Committee of Medical Journal Editors (ICMJE) Uniform Requirements for Manuscripts Submitted to Biomedical Journals - for further information please see here: https://journals.plos.org/plosone/s/authorship.

Yes, everyone listed as author meet PLOS' criteria for authorship.

Human subjects research (e.g. health research, medical research, cross-cultural psychology)

Did you obtain written informed consent from a representative of the local community or region before the research took place? How did you establish who speaks for the community? Details of written informed consent obtained from study participants should be reported separately in the Methods section of your manuscript.

The guidelines needed for each institution or louse collection center where lice were collected were followed. Since there is a large number of sites and collectors, plus some of the collections were done in the 2000's, we don't have all the list of institutions that provided permits. However, in all cases the collection of lice was voluntary and no personal information was collected. In case of minors, a parent or legal custodian provided consent. Before the louse collection, each person was informed about the purpose of the research and any questions that they have were answered. Because, UF waived the needed of written consent, the consents of the participants were not recordered, and the only information associated with each louse was the geographic location.

How did members of the local community provide input on the aims of the research investigation, its methodology, and its anticipated outcome(s)?

For our current research on human louse evolution, the local communities did not provide inputs about the aims of the research investigation or methodology. However, the gained knowledge will be shared widely through this open access publication so it can reach the whole world. In those places where it is possible to go back, we will go back and provide the article to the institutions that participated in the research in person, or if it is not possible in person, via email.

When engaging with the local community, how did you ensure that the informed consent documents and other materials could be understood by local stakeholders?

Because our collectors were native from the region or knew local language, the information was translated to their local language to ensure that each volunteer participant understood the louse collection protocol and consent on it.

Will the findings of the research be made available in an understandable format to stakeholders in the community where the study was conducted (e.g. via a presentation, summary report, copies of publications, etc.)? Please provide details of how this will be achieved.

This will be the first publication of this research, local presentations in Argentina and Mexico by two of the co-authors have been already been conducted. As opportunities arise, new local presentations in areas of collection will also been done. A copy of the current manuscript will also be provided to the institutions that participated in person or via email.

Non-human subjects research using specimens/ animals collected as part of the study, or those housed in archival collections. Examples include archaeology, paleontology, botany and zoology.

Did the permission you obtained from a local authority to perform the study include an agreement on access to outputs and benefit sharing? This may include procedures to enable fair distribution of the benefits and resources arising from the research performed. Please include any details of Prior Informed Consent and Benefit Sharing Agreements obtained. These may be required by field-specific regulations, for example the Convention on Biological Diversity (CBD) and the associated Nagoya Protocol.

The gained knowledge in this research will be shared widely through this open access publication so it can reach the whole world. All data will be publicy available as well.

If the material used in your study was imported, please A) provide the year it was imported and B) indicate whether permits were obtained to import/export the materials used, C) provide details of any permits obtained. If this information is not available, please indicate this.

We don’t have detailed dates when samples were shipped, however all the samples used in this study were shipped to the Reed Lab between 2005 to 2012. Importation permit was issued at that time.

If you used archival specimens, please state how the material used in your study was acquired by the institute it is held in and provide details of any permits obtained for the original excavations/ sample collection. If this information is not available, please indicate this.

N/A

How was the potential cultural significance of the materials collected in your study to local communities considered in your research design? Were Indigenous peoples and/or local researchers and institutions involved with archaeological excavations / collection of specimens? If so, please provide a description of their involvement.

N/A

If your manuscript includes photographs of human remains please indicate whether authors obtained permission from descendants or affiliated cultural communities to do so.

N/A

Supporting Information: World wide louse microsatellite data used for article: Nuclear genetic diversity of head lice sheds light on human dispersal around the world, Ascunce et. al. (2023), PONE-D-22-28588

		Loci																													
**Countries**	**Sites**	**T8_1F**		**M3_10**		**M3_19**		**M2_2PT**		**T2_6**		**M2_19**		**M2_13**		**M2_3**		**T6_11**		**T10_3v2**		**T9_4**		**T9_6**		**T2_7**		**T4_5v2PT**		**T1_4v2PT**	
Rwanda	Rw	119	127	265	271	349	349	400	408	120	120	189	193	295	299	372	376	117	117	217	217	160	168	124	124	210	210	241	241	198	216
Israel	Isr	119	123	271	271	?	?	402	402	?	?	191	193	297	301	382	382	117	117	?	?	160	160	116	124	202	202	241	241	218	220
		119	123	271	271	?	?	402	402	?	?	189	191	295	299	376	380	117	117	?	?	160	160	112	120	204	204	241	241	218	218
Nepal	Np	119	119	268	274	349	352	406	410	116	116	191	203	307	311	368	370	117	129	227	233	168	168	116	116	208	212	245	251	218	218
Laos	Ls	111	111	280	280	349	349	416	420	104	104	187	187	297	299	368	370	117	117	241	241	164	164	116	116	208	208	249	251	218	218
		115	123	283	283	349	349	416	416	104	104	209	211	?	?	370	372	117	121	227	237	164	164	116	116	208	208	249	249	218	220
		111	111	274	274	343	349	402	420	100	100	191	191	299	299	376	376	129	129	231	233	160	160	112	112	208	208	251	251	214	214
		111	119	271	280	349	349	420	424	116	116	187	187	297	297	372	390	125	125	231	233	160	160	112	116	208	208	245	251	218	218
Thailand	Th	107	107	274	280	355	355	404	412	108	116	189	189	295	295	374	374	125	125	227	235	160	160	116	120	208	208	245	245	218	218
Cambodia	Cam	111	119	268	295	364	364	412	430	104	108	173	173	?	?	366	378	125	125	237	247	160	160	116	116	208	208	237	241	216	218
		119	119	271	289	352	355	410	410	116	124	187	187	295	303	366	374	117	125	219	219	156	160	116	116	208	208	?	?	212	218
		103	119	268	268	346	355	412	414	108	116	189	199	297	297	378	380	121	125	233	237	156	160	116	116	208	208	241	241	214	218
		111	119	268	280	352	352	416	416	104	104	217	217	291	307	366	366	129	129	219	219	168	168	120	120	204	204	?	?	218	218
		115	145	271	271	352	352	402	412	104	112	173	173	297	297	374	378	121	121	233	237	164	164	116	116	208	212	251	251	216	216
		103	107	280	280	352	355	410	416	116	116	189	193	307	311	366	374	117	125	225	251	160	164	116	116	204	208	249	249	218	218
		107	119	268	283	346	352	410	412	112	112	173	187	297	297	374	378	125	125	237	247	156	160	116	116	208	208	245	245	218	218
		115	145	271	271	346	352	410	424	116	116	187	187	?	?	372	374	121	125	225	225	160	168	116	116	204	208	261	261	218	218
		111	119	268	280	352	364	410	410	116	116	173	173	293	299	374	378	125	129	225	225	164	164	116	116	204	204	?	?	218	218
		103	107	271	271	355	355	?	?	112	116	187	189	295	295	366	366	121	125	231	231	160	164	116	116	204	208	245	245	218	218
		111	119	280	280	352	361	412	412	108	112	173	173	291	295	366	374	125	129	225	233	160	168	116	120	208	208	237	237	214	218
		115	115	265	265	346	346	410	410	104	116	173	173	?	?	370	370	113	129	221	221	160	164	116	116	208	208	237	237	218	218
		103	111	283	283	352	364	406	412	104	116	173	173	?	?	366	372	129	129	225	225	160	160	116	116	208	208	243	243	218	218
		111	119	271	271	352	364	412	414	104	112	173	173	?	?	366	370	125	129	219	247	160	168	116	116	208	208	249	249	210	218
		111	119	283	283	364	364	406	412	104	112	173	187	?	?	370	374	125	125	233	247	164	164	116	116	204	208	245	257	218	218
		107	111	271	280	352	355	410	410	116	116	193	217	307	307	366	366	117	129	219	225	164	168	116	116	204	208	249	249	214	218
		133	133	268	268	352	352	410	410	108	108	185	185	?	?	376	376	117	117	239	239	160	160	116	116	204	204	243	243	220	220
		111	111	265	265	355	355	412	418	116	116	187	187	?	?	366	370	117	125	219	225	160	164	116	116	208	208	237	249	218	218
		107	111	265	265	355	355	410	418	116	116	193	199	?	?	366	372	117	125	219	227	164	168	116	116	208	208	237	249	218	218
		111	123	286	286	352	358	402	414	116	116	201	201	?	?	378	378	125	125	219	227	160	164	116	116	208	208	237	243	218	218
		111	111	277	277	355	383	412	414	116	116	187	201	?	?	370	372	121	125	227	241	160	160	116	116	204	208	239	239	208	218
		107	111	277	286	352	383	412	418	116	116	187	189	297	297	372	380	129	129	241	241	160	164	116	116	204	208	237	237	214	218
Mongolia	Mn	119	123	262	271	349	349	400	424	112	116	185	185	305	305	378	380	117	121	229	229	160	172	112	116	204	208	245	251	210	222
		119	119	268	271	358	358	414	414	128	128	199	199	295	309	378	382	125	125	229	229	160	168	112	120	208	208	249	249	220	220
		119	119	268	271	358	358	414	414	128	128	199	199	295	309	378	382	117	125	229	229	160	168	112	120	208	208	249	249	220	220
		119	119	268	271	358	358	414	414	128	128	199	199	309	309	378	382	117	125	229	229	160	168	112	120	196	208	249	249	220	220
		119	123	268	271	358	358	414	414	128	128	199	199	295	309	382	382	117	125	229	229	160	168	112	120	196	208	249	249	220	220
		111	119	268	274	352	352	404	404	116	132	195	199	295	309	366	366	117	117	227	227	160	164	120	120	206	206	245	251	210	214
		111	111	271	274	343	352	406	406	112	112	189	205	295	303	370	374	117	117	219	219	160	164	116	116	208	208	241	249	218	220
		119	123	274	277	349	364	402	416	124	136	191	199	297	297	370	370	117	121	235	235	160	168	112	112	216	216	?	?	220	220
		119	123	274	277	364	364	416	416	136	136	191	199	?	?	370	374	117	133	235	235	160	164	112	116	202	214	247	247	220	220
		119	123	274	277	349	364	402	416	124	136	191	199	?	?	370	370	117	133	235	235	160	164	112	116	202	216	247	247	220	220
Turkey	Tur	115	115	268	268	355	355	416	416	122	122	203	205	295	295	374	374	125	125	227	227	160	160	116	116	204	204	243	243	220	220
		111	111	268	271	355	358	398	428	108	128	185	189	295	295	374	374	125	125	227	227	160	160	112	120	204	222	255	255	220	220
		115	115	268	268	355	355	416	416	122	122	189	203	295	295	374	374	125	125	227	227	160	160	120	128	204	204	243	243	220	220
		111	111	268	271	355	355	410	412	116	116	195	195	295	295	370	374	125	125	227	235	160	160	116	116	204	204	251	251	220	220
		115	115	268	268	355	355	416	416	108	122	185	203	295	295	374	376	125	125	227	235	160	160	120	120	204	204	243	243	220	220
		115	115	268	268	355	355	400	400	122	122	189	203	295	295	376	376	125	125	227	227	160	160	112	112	204	204	241	243	220	220
		133	133	268	268	355	355	416	416	122	122	189	189	303	303	374	374	125	125	227	227	160	160	120	128	204	204	243	243	220	220
		133	133	268	268	355	355	416	416	108	108	189	189	295	295	376	376	125	125	235	235	160	160	112	116	204	204	243	243	220	220

		Loci																													
**Countries**	**Sites**	**T8_1F**		**M3_10**		**M3_19**		**M2_2PT**		**T2_6**		**M2_19**		**M2_13**		**M2_3**		**T6_11**		**T10_3v2**		**T9_4**		**T9_6**		**T2_7**		**T4_5v2PT**		**T1_4v2PT**	
		111	115	268	268	355	355	416	428	128	128	185	189	295	295	374	374	125	125	?	?	?	?	112	116	204	204	243	243	220	220
		115	123	268	268	355	355	416	416	122	122	203	205	303	303	376	376	125	125	227	227	160	160	112	120	204	204	243	243	220	220
		115	133	268	268	355	355	416	416	122	122	203	203	295	295	374	376	125	125	227	227	160	160	120	128	204	204	243	243	206	220
		115	123	268	268	355	355	406	416	122	122	185	185	295	295	376	376	125	125	227	227	160	160	120	128	204	204	243	243	220	220
Croatia	Croa	115	115	268	268	355	355	416	416	108	108	189	189	295	295	374	374	125	125	227	227	160	160	116	120	204	204	243	243	220	220
		115	115	268	268	355	355	416	416	108	108	189	189	295	295	374	374	125	125	227	227	160	160	116	120	204	204	243	243	220	220
		115	115	268	268	355	355	398	416	108	108	189	189	?	?	374	374	125	125	227	227	160	160	120	120	204	204	243	243	220	220
		115	115	268	268	?	?	?	?	108	108	189	189	295	295	374	374	125	125	?	?	160	160	116	116	204	204	243	243	220	220
		115	115	268	268	355	355	398	416	108	108	189	189	295	295	374	374	125	125	227	227	160	160	116	116	204	204	243	243	220	220
		115	115	268	268	355	355	416	416	108	108	189	189	295	295	374	374	125	125	227	227	160	160	116	120	204	204	243	243	220	220
Norway	Nw	115	115	268	268	355	355	416	416	100	108	?	?	295	295	376	376	125	125	227	227	160	160	116	116	204	204	243	243	218	220
Netherlands	Neth	133	133	268	268	355	355	398	398	122	122	185	185	295	295	374	374	125	125	227	227	160	160	120	120	204	204	243	243	220	220
		133	133	268	268	355	355	398	398	122	122	185	185	295	295	374	374	125	125	227	227	160	160	120	120	204	204	243	243	220	220
		133	133	268	268	355	355	398	398	122	122	185	185	295	295	374	374	125	125	227	227	160	160	120	120	204	204	243	243	220	220
		133	133	268	268	355	355	398	398	122	122	185	185	295	295	374	374	125	125	227	227	160	160	120	120	204	204	243	243	220	220
		133	133	268	268	355	355	398	398	122	122	185	185	295	295	374	374	125	125	227	227	160	160	120	120	204	204	243	243	220	220
		133	133	268	268	355	355	398	398	122	122	185	185	295	295	374	374	125	125	227	227	160	160	120	120	204	204	243	243	220	220
		133	133	268	268	355	355	398	398	108	108	189	189	295	295	374	374	125	125	227	227	160	160	120	120	204	204	243	243	220	220
		133	133	268	268	355	355	398	398	100	122	185	185	295	295	374	376	125	125	227	227	160	160	116	116	204	204	243	243	220	220
		133	133	268	268	355	355	416	416	100	122	185	185	295	295	368	368	125	125	227	227	160	160	120	120	204	204	243	243	220	220
		133	133	268	268	355	355	416	416	108	108	185	185	297	297	374	374	125	125	227	227	160	160	120	120	204	204	243	243	220	220
		133	133	268	268	355	355	416	416	108	108	185	185	293	301	374	374	125	125	227	227	160	160	120	120	204	204	243	243	220	220
		133	133	268	268	355	355	416	416	108	108	185	185	297	301	374	374	125	125	227	227	160	160	120	120	204	204	243	243	220	220
		133	133	268	268	355	355	416	416	108	108	185	185	297	301	374	374	125	125	227	227	160	160	120	120	204	204	243	243	220	220
		133	133	268	268	355	355	416	416	108	108	185	185	301	301	374	374	125	125	227	227	160	160	120	120	204	204	243	243	220	220
		133	135	268	268	355	355	416	416	108	108	185	185	301	301	374	374	125	125	227	227	160	160	116	120	204	204	243	243	206	220
		133	133	268	268	355	355	416	416	108	108	185	185	301	301	374	374	125	125	227	227	160	160	120	120	204	204	243	243	220	220
		133	133	268	268	355	355	416	416	108	108	185	185	301	301	374	374	125	125	227	227	?	?	120	120	204	204	243	243	220	220
		133	133	268	268	355	355	416	416	108	108	185	185	301	301	374	374	125	125	227	227	160	160	120	120	204	204	243	243	220	220
		133	133	268	268	355	355	416	416	108	108	185	185	301	301	374	374	125	125	227	227	160	160	120	120	204	204	243	243	220	220
		133	133	268	268	355	355	416	416	108	108	185	185	301	301	374	374	125	125	227	227	160	160	120	120	204	204	243	243	220	220
		133	133	268	268	355	355	416	416	108	108	185	185	301	301	374	374	125	125	227	227	160	160	120	120	204	204	243	243	220	220
		133	133	268	268	355	355	416	416	108	108	185	185	?	?	374	374	125	125	227	227	160	160	120	120	204	204	243	243	220	220
		133	133	268	268	355	355	416	416	108	108	185	185	301	301	374	374	125	125	227	227	160	160	120	120	204	204	243	243	220	220
		133	133	268	268	355	355	416	416	108	108	185	185	301	301	374	374	125	125	227	227	160	160	120	120	204	204	243	243	220	220
		133	133	268	268	355	355	416	416	108	108	185	185	301	301	374	374	125	125	227	227	160	160	120	120	204	204	243	243	220	220
		133	133	268	268	355	355	416	416	108	108	185	185	?	?	370	374	125	125	227	227	160	160	120	120	204	204	243	243	220	220
		133	133	268	268	355	355	416	416	108	108	185	185	?	?	374	374	125	125	227	227	160	160	120	120	204	204	243	243	220	220
		133	133	268	268	355	355	416	416	108	108	185	185	301	301	374	374	125	125	227	227	160	160	120	120	204	204	243	243	220	220
		133	133	268	268	355	355	416	422	108	108	185	185	301	301	374	374	125	125	227	227	160	160	120	120	204	204	243	243	220	220
		133	133	268	268	355	355	416	416	108	108	185	185	301	301	374	374	125	125	227	227	160	160	120	120	204	204	243	243	220	220
		133	133	268	268	355	355	416	416	108	108	185	185	301	301	374	374	125	125	227	227	160	160	120	120	204	204	243	243	220	220
		133	133	268	268	355	355	416	416	108	108	185	185	301	301	374	374	125	125	227	227	160	160	120	120	204	204	243	243	220	220
		133	133	268	268	355	355	416	416	108	108	185	185	301	301	374	374	125	125	227	227	160	160	120	120	204	204	243	243	220	220
		133	133	268	268	355	355	416	416	108	108	185	185	301	301	374	374	125	125	227	227	160	160	120	120	204	204	243	243	220	220
		133	133	268	268	355	355	416	416	108	108	185	185	?	?	374	374	125	125	227	227	160	160	120	120	204	204	243	243	220	220
		133	133	268	268	355	355	416	416	108	108	185	185	?	?	374	374	125	125	227	227	160	160	120	120	204	204	243	243	220	220
		133	133	268	268	355	355	416	416	108	108	185	185	301	301	374	374	125	125	227	227	160	160	120	120	204	204	243	243	220	220
		133	133	268	268	355	355	416	416	108	108	185	185	301	301	374	374	125	125	227	227	160	160	120	120	204	204	243	243	220	220

Supporting Information: World wide louse microsatellite data used for article: Nuclear genetic diversity of head lice sheds light on human dispersal around the world, Ascunce et. al. (2023), PONE-D-22-28588

		Loci																											
**Countries**	**Sites**	**T8_1F**		**M3_10**		**M3_19**		**M2_2PT**		**T2_6**		**M2_19**		**M2_13**		**M2_3**		**T6_11**		**T10_3v2**		**T9_4**		**T9_6**		**T2_7**		**T4_5v2PT**		**T1_4v2PT**	
		133	133	268	268	355	355	416	416	108	108	185	185	301	301	374	374	125	125	227	227	160	160	120	120	204	204	243	243	220	220
		133	133	268	268	355	355	416	416	108	108	185	185	301	301	374	374	125	125	227	227	160	160	120	120	204	204	243	243	220	220
		133	133	268	268	355	355	416	416	108	108	185	185	?	?	374	374	125	125	227	227	160	160	120	120	204	204	243	243	220	220
		133	133	268	268	355	355	416	416	108	108	185	185	?	?	374	374	125	125	227	227	160	160	120	120	204	204	243	243	220	220
		133	133	268	268	355	355	416	416	100	100	185	185	301	301	374	374	125	125	227	227	160	160	120	120	204	204	243	243	218	218
		133	133	268	268	355	355	416	416	100	100	185	185	301	301	374	374	125	125	227	227	160	160	120	120	204	204	243	243	218	218
		133	133	268	268	355	355	416	416	100	100	185	185	?	?	374	374	125	125	227	227	160	160	120	120	204	204	243	243	218	218
		133	133	268	299	355	355	416	416	100	100	189	189	301	301	376	376	117	125	227	227	160	160	116	116	204	204	243	243	220	220
		115	115	299	299	355	355	398	398	100	100	185	185	295	295	374	374	125	125	227	227	160	160	116	116	204	204	243	243	218	218
		115	115	299	299	355	355	398	398	100	100	185	185	295	295	374	374	125	125	227	227	160	160	116	116	204	204	243	243	218	218
		115	133	268	268	355	355	416	416	100	108	185	185	295	295	374	374	125	125	227	227	160	160	120	120	204	204	243	243	220	220
		133	133	268	268	355	355	416	416	108	108	185	185	295	295	374	374	125	125	227	227	160	160	120	120	204	204	243	243	220	220
		115	115	268	268	355	355	416	416	108	108	185	185	295	295	374	374	125	125	227	227	160	160	120	120	204	204	243	243	220	220
		133	133	268	268	355	355	416	416	100	100	185	185	295	295	374	374	125	125	227	227	160	160	120	120	204	204	243	243	220	220
		133	133	268	268	355	355	416	416	100	108	185	185	295	295	374	374	125	125	227	227	160	160	120	120	204	204	243	243	220	220
		133	133	268	268	355	355	416	416	100	100	185	185	301	301	374	374	125	125	227	227	160	160	120	120	204	204	243	243	220	220
		133	133	268	268	355	355	416	416	100	100	185	185	301	301	374	374	125	125	227	227	160	160	120	120	204	204	243	243	220	220
		133	133	299	299	355	355	416	416	100	100	189	189	295	295	374	374	125	125	227	227	160	160	120	120	204	204	243	243	220	220
		133	133	268	268	355	355	398	398	100	100	189	189	295	295	374	376	125	125	227	227	160	160	120	120	204	204	243	243	220	220
		133	133	299	299	355	355	416	416	100	122	185	185	295	295	374	374	125	125	227	227	160	160	120	120	204	204	243	243	220	220
		133	133	268	268	355	355	408	416	122	122	189	189	295	295	374	374	125	125	227	227	160	160	120	120	204	204	243	243	220	220
		133	133	268	268	355	355	408	416	122	122	189	189	295	295	374	374	125	125	227	227	160	160	120	120	204	204	243	243	220	220
		133	133	268	268	355	355	408	408	122	122	189	189	295	295	374	374	125	125	227	227	160	160	120	120	204	204	243	243	220	220
		133	133	268	268	355	355	416	416	100	100	185	185	?	?	374	374	125	125	227	227	160	160	120	120	204	204	243	243	220	220
		115	115	268	268	355	355	416	416	108	108	189	189	?	?	376	376	125	125	227	227	160	160	116	116	204	204	243	243	220	220
		133	133	268	268	355	355	416	416	122	122	189	189	295	295	376	376	125	125	227	227	160	160	120	120	204	204	243	243	220	220
France	Fr	133	133	268	268	355	355	398	416	108	108	189	189	295	295	376	376	125	125	227	227	160	160	116	116	204	204	243	243	220	220
		133	133	268	268	355	355	398	416	108	108	189	189	295	295	376	376	117	125	227	227	160	160	116	116	204	204	243	243	220	220
		133	133	268	268	355	355	398	416	108	108	189	189	295	295	376	376	125	125	227	227	160	160	116	116	204	204	243	243	220	220
United Kingdom	UK	115	133	268	268	355	355	398	398	108	108	189	189	295	295	376	376	125	129	?	?	?	?	120	120	204	204	243	243	220	220
		115	115	268	268	355	355	398	398	108	108	189	189	295	295	374	376	125	125	235	235	160	160	120	120	204	204	243	243	220	220
		115	133	268	268	355	355	398	398	108	108	189	189	295	295	376	376	125	125	235	235	160	160	120	120	204	204	243	243	220	220
		115	115	268	268	355	355	398	398	108	108	189	189	295	295	376	376	125	125	235	235	160	160	120	120	204	204	243	243	220	220
		133	133	268	268	355	355	398	398	108	108	189	189	295	295	376	376	125	125	235	235	160	160	120	120	204	204	243	243	220	220
		133	133	265	268	355	355	416	416	108	108	189	191	295	295	374	376	121	121	?	?	?	?	120	120	204	204	243	243	220	220
		133	133	265	268	355	355	416	416	108	112	189	191	295	295	374	376	121	121	?	?	?	?	116	120	204	204	243	243	220	220
		115	115	268	268	355	355	408	408	108	108	189	189	295	295	376	376	125	125	227	227	160	160	116	124	204	204	243	243	220	220
		133	133	268	268	355	355	416	416	100	108	191	191	295	295	378	378	125	125	227	227	160	168	112	116	204	204	243	243	220	220
		133	133	265	268	355	355	416	416	108	108	191	191	295	295	374	374	125	125	221	235	160	160	116	116	204	204	243	243	220	220
		133	133	265	268	355	355	416	416	108	112	189	191	295	295	374	374	125	125	221	227	160	160	120	120	204	204	243	245	220	220
		115	133	268	268	355	355	398	398	108	108	189	189	295	295	374	376	125	125	235	235	160	160	120	120	204	204	243	243	220	220
		115	133	268	268	355	355	398	416	108	128	189	191	295	295	374	376	125	125	227	227	160	160	120	120	204	204	243	243	220	220
		133	133	268	268	355	355	416	416	108	108	191	191	295	295	374	376	125	125	227	227	160	160	120	120	204	204	243	243	220	220
		115	115	268	268	355	355	408	408	108	108	189	189	295	295	376	376	125	125	227	227	160	160	?	?	204	204	243	243	220	220
		133	133	268	268	355	355	416	416	100	100	189	189	?	?	376	376	125	125	227	227	160	160	120	120	204	204	243	243	220	220
		133	133	268	268	355	355	416	416	100	100	189	189	?	?	376	376	125	125	227	227	160	160	120	120	204	204	243	243	220	220
		133	133	268	268	355	355	416	416	108	108	189	189	295	295	374	374	125	125	227	227	160	160	112	128	204	204	243	243	220	220
		115	115	268	268	355	355	416	416	108	108	189	189	295	295	376	376	125	125	227	227	160	160	?	?	204	204	243	243	220	220
		115	115	268	268	355	355	398	398	100	108	191	191	295	295	374	374	125	125	227	227	160	160	112	116	204	204	243	243	218	220

Supporting Information: World wide louse microsatellite data used for article: Nuclear genetic diversity of head lice sheds light on human dispersal around the world, Ascunce et. al. (2023), PONE-D-22-28588

		Loci																															
**Countries**	**Sites**	**T8_1F**		**M3_10**		**M3_19**		**M2_2PT**		**T2_6**		**M2_19**		**M2_13**		**M2_3**		**T6_11**		**T10_3v2**		**T9_4**		**T9_6**		**T2_7**		**T4_5v2PT**		**T1_4v2PT**	
		115	115	268	268	355	355	416	416	108	108	189	191	295	295	376	376	125	125	227	227	160	160	?	?	204	204	243	243	220	220
		115	133	268	268	355	355	410	410	108	122	189	189	295	295	374	374	125	125	227	227	160	160	120	120	204	204	243	243	220	220
		133	133	268	268	355	355	410	410	122	122	189	189	295	295	374	374	125	125	227	227	160	160	120	120	204	204	243	243	220	220
		115	133	268	268	355	355	410	410	122	122	189	189	295	295	374	376	125	125	227	227	160	160	120	120	204	204	243	243	220	220
		133	133	268	268	355	355	410	410	122	122	189	189	295	295	374	374	125	125	227	227	160	160	120	120	204	204	243	243	220	220
		115	115	268	268	355	355	416	416	100	108	189	189	295	295	374	376	125	125	227	227	160	160	116	116	204	204	243	243	220	220
		133	133	268	268	355	355	398	416	100	108	191	191	295	295	374	376	125	125	227	235	160	160	120	120	204	204	243	243	220	220
Spain	Spain	123	133	268	271	355	355	410	410	100	100	189	189	295	295	376	376	117	125	227	227	160	160	108	116	204	204	243	253	220	220
		119	123	268	271	355	355	404	410	100	112	189	191	295	295	376	376	117	125	227	235	160	160	108	108	204	204	245	253	220	220
		119	123	271	271	?	?	402	416	?	?	189	191	295	295	374	376	117	117	?	?	160	160	108	108	204	204	239	245	216	220
USA	Oce	115	115	268	268	355	355	398	398	100	100	189	189	295	295	374	374	125	125	227	227	160	160	120	120	204	204	243	243	218	243
		133	133	268	268	355	355	416	416	100	100	189	189	295	295	374	376	117	125	227	227	160	160	120	120	204	204	243	243	220	220
		115	115	268	268	355	355	416	416	100	100	189	189	295	295	376	376	125	125	227	227	160	160	116	120	204	204	243	243	218	220
		133	133	268	268	355	355	416	416	100	100	185	185	301	301	374	374	125	125	227	227	160	160	120	120	204	204	243	243	220	220
		115	115	268	268	355	355	418	418	100	108	189	189	295	295	376	376	117	125	227	227	160	160	120	120	204	204	243	243	220	220
		115	115	268	271	355	355	398	398	100	108	189	189	295	297	376	376	125	125	227	227	160	160	120	120	204	204	243	243	220	220
		115	115	268	268	355	355	398	398	100	100	189	189	291	309	376	376	125	125	227	227	160	160	120	120	204	204	243	243	220	220
		133	133	268	268	355	355	416	416	108	108	189	189	301	301	374	374	125	125	227	227	160	160	120	120	204	204	243	243	218	218
		115	115	268	268	355	355	406	406	108	108	189	189	299	299	374	374	125	125	227	227	160	160	120	120	204	204	243	243	220	220
		115	115	268	268	355	355	398	398	100	100	189	189	291	299	376	376	125	125	227	227	160	160	120	120	204	204	243	243	220	220
		133	133	274	274	352	352	398	398	122	122	203	205	295	303	374	374	125	125	227	227	160	160	116	116	204	204	239	239	220	220
		115	115	268	268	355	355	398	398	100	100	185	189	295	295	374	374	125	125	227	227	160	160	120	120	204	204	243	243	218	218
		133	133	268	268	355	355	416	416	122	122	185	185	295	295	374	374	117	125	227	227	160	160	120	120	204	204	243	243	220	220
		133	133	268	268	355	355	398	398	100	100	189	189	295	295	376	376	125	125	227	227	160	160	120	120	204	204	243	243	220	220
		133	133	268	268	355	355	416	416	100	100	185	185	291	301	374	374	125	125	227	227	160	160	120	120	204	204	243	243	220	220
		115	115	268	268	355	355	398	398	100	100	189	189	295	295	374	374	125	125	227	227	160	160	120	120	204	204	243	243	218	218
		133	133	268	268	355	355	416	416	100	100	185	185	301	301	374	374	117	125	227	227	160	160	120	120	204	204	243	243	220	220
		133	133	268	268	355	355	416	416	100	100	185	185	301	301	374	374	125	125	227	227	160	160	120	120	204	204	243	243	220	220
		115	115	268	268	355	355	416	416	108	108	189	189	295	295	374	374	125	125	227	227	160	160	112	120	204	204	243	243	218	220
		115	115	268	268	355	355	416	416	100	100	189	189	295	295	376	376	125	125	227	227	160	160	120	120	204	204	243	243	220	220
		115	115	268	268	355	355	416	416	100	100	189	189	295	295	376	376	125	125	227	227	160	160	120	120	204	204	243	243	220	220
		133	133	268	268	355	355	416	416	108	108	189	189	295	295	376	376	125	125	227	227	160	160	112	112	204	204	243	243	220	220
		133	133	268	268	355	355	416	416	100	100	185	185	297	301	374	374	117	125	227	227	160	160	120	120	204	204	243	243	220	220
		115	115	268	268	355	377	416	416	100	100	189	189	295	295	376	376	125	125	227	227	160	160	120	120	204	204	243	243	220	220
		133	133	268	268	355	373	398	398	108	108	189	189	295	295	374	374	117	125	227	227	160	160	120	120	204	204	243	243	220	220
USA	SF	115	115	268	268	355	355	398	398	100	100	189	189	295	295	374	374	125	125	227	227	160	160	120	120	204	204	243	243	218	218
USA	Martin	133	133	268	268	355	355	414	414	100	100	?	?	295	295	376	376	117	117	227	227	160	160	120	120	204	204	243	243	220	220
USA	WPB	115	133	299	299	352	355	416	416	100	108	189	191	299	299	374	374	117	125	223	227	160	160	120	120	204	204	243	243	218	220
USA	Nash	115	115	268	268	355	355	416	416	100	100	189	189	303	303	374	374	125	125	227	227	160	160	120	120	204	204	243	243	220	220
		115	115	268	268	355	355	416	416	100	100	189	189	303	303	374	374	125	125	227	227	160	160	120	120	204	204	243	243	220	220
		115	115	268	268	355	355	416	416	100	100	189	189	303	303	374	374	125	125	227	227	160	160	120	120	204	204	243	243	220	220
		107	107	268	268	355	355	416	416	100	100	189	189	295	295	376	376	125	125	227	227	160	160	120	120	204	204	243	243	220	220
		107	107	268	268	355	355	416	416	100	100	189	189	295	295	376	376	125	125	227	227	160	160	120	120	204	204	243	243	220	220
		133	133	268	268	355	355	416	416	100	100	185	185	301	301	374	374	125	125	227	227	160	160	120	120	204	204	243	243	220	220
		133	133	268	268	355	355	416	416	100	100	185	185	301	301	374	374	125	125	227	227	160	160	120	120	204	204	243	243	220	220
		107	107	268	268	355	355	416	416	100	100	189	189	295	295	376	376	125	125	227	227	160	160	120	120	204	204	243	243	220	220
		115	115	268	268	355	355	410	410	122	122	203	205	295	295	376	376	125	125	249	249	164	164	112	116	204	204	243	243	220	220

Supporting Information: World wide louse microsatellite data used for article: Nuclear genetic diversity of head lice sheds light on human dispersal around the world, Ascunce et. al. (2023), PONE-D-22-28588

		Loci																													
Countries	Sites	T8_1F		M3_10		M3_19		M2_2PT		T2_6		M2_19		M2_13		M2_3		T6_11		T10_3v2		T9_4		T9_6		T2_7		T4_5v2PT		T1_4v2PT	
		107	107	268	268	355	355	416	416	100	100	189	189	295	295	376	376	125	125	227	227	160	160	120	120	204	204	243	243	220	220
		107	107	268	268	355	355	416	416	100	100	189	189	295	295	376	376	125	125	227	227	160	160	120	120	204	204	243	243	220	220
		133	133	268	271	355	361	398	398	122	122	189	189	295	295	376	376	125	125	227	227	160	160	120	120	204	204	243	243	220	220
		107	133	268	268	355	355	416	416	108	108	189	189	295	295	376	376	125	125	227	227	160	160	116	120	204	204	243	243	220	220
		115	115	299	299	355	355	398	398	108	108	203	203	295	295	370	376	125	125	227	227	160	160	120	120	204	204	243	243	218	218
		115	115	268	268	355	355	448	448	100	100	185	185	303	303	374	374	125	125	227	227	160	160	120	120	204	204	243	243	218	218
		115	115	268	268	355	355	416	416	100	100	189	189	?	?	374	374	125	125	227	227	160	160	120	120	204	204	243	243	220	220
		107	107	268	268	355	355	416	416	100	100	189	189	295	295	376	376	125	125	227	227	160	160	120	120	204	204	243	243	220	220
		107	107	268	268	355	355	416	416	100	100	189	189	295	295	374	376	125	125	227	227	160	160	120	120	204	204	243	243	220	220
USA	MC	107	107	268	268	355	355	416	416	100	108	185	185	295	295	376	376	117	117	231	231	160	160	116	116	204	204	243	243	220	220
		115	115	268	268	355	355	416	416	100	100	189	189	295	295	376	376	117	125	227	227	160	160	116	116	204	204	243	243	220	220
		107	107	268	268	355	355	416	416	100	100	189	189	295	295	376	376	117	125	227	227	160	160	120	120	204	204	243	243	220	220
		107	107	268	268	355	355	416	416	100	100	189	189	295	295	376	376	117	125	227	227	160	160	120	120	204	204	243	243	220	220
		133	133	268	268	355	355	406	416	128	128	203	205	309	309	376	376	117	133	227	227	160	164	112	116	204	204	243	243	220	220
		103	133	268	268	355	355	406	406	?	?	203	205	309	309	376	376	117	133	227	227	160	164	112	116	204	204	243	243	220	220
		115	115	268	268	355	355	416	416	100	100	189	189	295	295	374	376	117	117	227	227	160	160	116	116	204	204	243	243	220	220
		115	115	268	268	355	355	416	416	100	100	185	189	295	295	376	376	117	117	227	227	160	160	116	116	204	204	243	243	220	220
		111	115	268	268	355	355	430	464	92	100	185	189	295	295	374	376	121	125	239	239	160	164	116	120	204	204	243	243	216	220
		115	115	274	274	355	355	416	416	100	122	199	199	305	305	374	374	117	125	227	233	160	164	120	120	204	204	243	243	218	218
		107	107	268	268	355	355	416	416	100	100	189	189	295	295	376	376	125	125	227	227	160	160	120	120	204	204	243	243	220	220
		115	115	268	268	355	355	416	416	100	100	189	189	295	295	376	376	117	117	227	227	160	160	116	116	204	204	243	243	220	220
		115	115	268	268	355	355	416	416	100	100	185	189	301	301	374	374	125	125	227	227	160	160	120	120	204	204	243	243	220	220
		133	133	268	268	355	355	416	416	122	122	185	185	295	295	374	374	125	125	227	227	160	160	120	120	204	204	243	243	220	220
		127	133	268	280	355	377	398	416	100	122	203	205	295	301	370	376	117	125	215	215	160	164	120	120	204	204	243	243	220	220
		115	115	280	280	355	355	398	416	122	122	189	203	301	301	370	370	117	125	215	215	160	164	116	116	204	204	243	243	220	220
		115	115	268	280	355	355	398	416	122	122	189	189	301	301	370	370	117	125	215	215	160	160	116	120	204	204	243	243	220	220
		103	107	268	268	355	355	416	416	100	100	189	189	295	295	376	376	125	125	227	227	160	160	120	120	204	204	243	243	220	220
		115	115	289	289	355	355	418	418	100	100	185	185	295	295	374	374	117	125	227	227	160	160	116	116	204	204	243	243	220	220
		133	133	?	?	355	355	416	418	108	108	193	193	?	?	376	376	117	125	227	227	160	160	116	116	204	204	239	239	220	220
		133	133	274	274	355	355	416	416	108	108	193	193	309	309	376	376	125	125	227	227	160	160	116	116	204	204	239	239	220	220
		115	133	299	299	355	355	446	446	100	100	185	185	309	309	374	374	125	125	227	227	160	160	120	120	204	204	243	243	218	218
Mexico	MX-1	133	133	268	268	355	355	412	416	108	108	189	189	?	?	376	378	125	125	227	256	160	160	120	120	204	204	243	243	220	220
		115	133	299	299	355	355	408	408	108	112	189	189	?	?	374	374	125	125	227	227	160	160	116	116	204	204	243	243	216	216
		133	133	268	268	355	355	412	416	108	112	189	189	?	?	376	376	125	125	227	227	160	160	132	132	204	204	243	243	220	220
		133	133	268	268	355	355	416	416	108	108	189	189	?	?	376	376	125	125	227	227	160	160	112	112	204	204	243	243	220	220
		133	133	268	268	355	355	408	416	108	108	189	201	?	?	376	382	125	125	227	227	160	160	112	112	204	204	243	243	216	220
		115	133	268	268	355	355	412	416	108	108	201	201	?	?	376	376	125	125	227	227	160	160	132	132	204	204	243	243	216	220
		133	133	268	268	355	355	416	416	104	108	189	201	?	?	376	376	125	125	227	227	160	160	132	132	204	204	243	243	220	220
		133	133	268	268	355	355	412	416	108	108	189	201	295	295	376	376	125	125	227	227	160	160	120	120	204	204	243	243	216	220
		133	133	268	268	355	355	422	422	112	112	189	201	295	295	376	376	125	125	227	227	160	160	116	116	204	204	243	243	216	216
		133	133	268	268	355	355	410	416	100	108	189	189	295	295	374	374	125	125	227	237	160	160	116	116	204	204	243	243	216	220
		133	133	268	268	355	355	414	416	92	108	189	189	295	295	374	374	125	125	231	237	160	160	116	116	204	204	243	243	214	216
		133	133	268	268	355	355	414	416	108	108	189	189	295	295	376	376	125	125	227	227	160	160	120	120	204	204	243	243	220	220
		115	115	274	277	355	355	410	416	100	108	203	203	295	295	374	374	125	125	237	237	160	160	116	116	204	204	243	247	220	220
		115	133	268	268	355	355	412	412	108	116	191	191	295	295	376	376	125	125	227	227	160	160	120	120	204	204	243	243	216	216
		133	133	268	268	355	355	416	416	108	108	189	189	?	?	376	376	125	125	227	227	160	160	112	112	204	204	243	243	220	220
		115	133	268	268	355	355	416	422	112	112	201	201	?	?	376	376	125	125	227	227	160	160	116	116	204	204	243	243	216	220

		Loci																													
**Countries**	**Sites**	**T8_1F**		**M3_10**		**M3_19**		**M2_2PT**		**T2_6**		**M2_19**		**M2_13**		**M2_3**		**T6_11**		**T10_3v2**		**T9_4**		**T9_6**		**T2_7**		**T4_5v2PT**		**T1_4v2PT**	
		127	133	268	268	355	355	410	410	100	108	201	201	?	?	376	378	117	125	227	227	164	164	116	120	204	204	243	243	220	220
		115	115	268	268	355	355	412	416	108	112	201	201	295	295	376	376	125	125	227	227	160	160	120	120	204	204	243	243	216	216
Mexico	DF	133	133	268	268	355	355	410	410	108	108	189	189	295	295	374	374	125	125	227	227	160	160	?	?	204	204	243	243	220	220
Honduras	Hon	107	107	277	283	355	355	406	410	108	132	187	215	?	?	376	378	117	117	231	235	160	160	120	120	204	204	243	243	216	218
		107	107	292	292	352	355	406	406	108	120	215	215	?	?	376	376	125	125	231	245	160	160	116	120	204	204	243	243	216	216
		111	115	274	292	355	358	410	410	108	130	189	189	297	297	374	374	117	117	231	231	160	164	116	124	204	204	243	243	218	218
		107	107	277	292	355	355	406	420	108	120	189	189	?	?	378	378	117	117	245	245	164	164	136	136	204	204	243	243	216	216
		107	115	274	283	352	355	410	410	108	116	189	205	?	?	376	378	117	117	231	231	160	164	120	120	204	204	243	245	218	218
		107	107	292	292	355	355	406	410	108	108	189	189	299	299	376	376	117	125	231	245	160	160	116	116	204	204	241	243	216	216
		107	107	283	283	355	355	406	406	108	108	189	189	?	?	376	376	117	117	231	237	160	164	116	120	204	204	243	243	218	218
		115	115	283	283	355	355	406	410	120	120	205	205	?	?	374	382	117	117	233	237	160	160	?	?	204	204	243	243	218	218
		115	115	277	292	355	358	410	410	100	108	189	189	303	303	376	376	117	125	231	235	160	164	116	120	204	204	243	243	216	218
		107	107	274	274	355	355	410	410	108	108	201	205	?	?	374	378	117	117	231	245	160	160	116	116	204	204	243	243	216	218
		107	107	283	283	352	355	406	406	116	132	221	221	?	?	370	374	117	117	231	237	160	160	?	?	204	204	243	243	218	218
		107	115	274	274	355	355	410	410	108	120	201	201	299	299	374	378	117	117	227	231	160	160	116	116	204	204	243	243	216	216
		115	115	280	280	352	355	406	410	120	120	205	205	?	?	374	376	117	117	237	245	160	160	116	116	204	204	243	243	216	216
		107	115	283	292	355	355	410	410	108	108	205	215	299	299	376	378	117	117	227	231	160	164	120	120	204	204	243	245	216	218
		115	115	292	292	358	358	422	422	120	120	189	189	299	299	374	374	117	117	227	237	160	160	116	132	204	204	239	267	216	218
		107	107	274	280	355	355	406	406	120	120	221	221	299	299	378	378	117	125	237	245	160	164	116	116	204	204	243	243	216	218
		115	115	292	292	352	355	420	420	108	116	189	189	?	?	374	378	117	117	229	233	160	160	116	116	204	204	243	243	218	218
		107	107	283	283	352	355	410	420	108	108	201	201	?	?	382	382	117	117	233	237	164	164	116	120	204	204	243	243	218	224
		107	115	283	292	352	355	406	410	?	?	189	211	?	?	376	376	117	125	231	245	164	164	120	120	204	204	243	243	218	218
		115	115	280	292	355	355	410	410	108	108	205	221	?	?	374	376	117	125	245	245	160	160	116	116	204	204	243	247	218	220
Peru	Peru	103	115	?	?	352	355	412	412	96	108	189	193	?	?	374	374	117	117	239	239	152	164	120	120	204	204	243	243	216	218
Argentina	Arg	119	133	268	280	349	355	412	412	104	108	189	189	303	303	374	374	117	125	237	237	160	160	116	116	204	204	243	251	218	220
		103	103	268	283	355	355	416	416	108	108	189	189	303	303	374	376	117	121	227	227	160	160	116	120	204	204	243	243	216	216
		115	115	268	268	352	355	398	414	100	104	189	197	295	295	370	376	117	117	213	227	160	160	116	120	204	204	243	243	216	218
		103	103	268	268	355	355	416	416	100	108	189	189	301	303	376	376	121	121	227	235	160	160	116	120	204	204	243	243	216	216
		115	115	268	268	352	355	398	414	100	104	197	197	295	295	370	376	117	117	227	227	160	160	120	120	204	204	243	243	216	220
		103	103	283	283	355	355	416	416	108	108	189	189	301	301	376	376	117	121	237	237	160	160	116	120	204	204	243	243	216	220
		115	133	268	268	355	355	416	416	100	108	189	189	295	295	376	376	117	117	227	227	156	160	116	120	204	204	243	243	220	220
		115	133	299	299	355	355	408	416	122	122	185	189	?	?	374	376	117	117	227	227	160	160	116	120	198	204	243	243	216	220
		103	103	268	268	346	355	416	416	100	108	189	189	303	303	374	376	121	121	227	237	160	160	120	120	204	204	243	243	216	218
