# Supplementary material for: Nuclear genetic diversity of head lice sheds light on human dispersal around the world
Source: PLoS One. 2023 Nov 8;18(11):e0293409. doi: 10.1371/journal.pone.0293409 (PMC10631634; doi:10.1371/journal.pone.0293409)
Supplement: S2 File — Groups reflect their geographic distribution and STRUCTURE clustering: Africa-Israel-Spain (AIS), South and Southeast Asia (SAs), East Asia (EAs), Europe (E), North America (NA), Mesoamerica (MA), Central America (CA), and South America (SAm). S2_S3 Tables. Prior distributions of population sizes parameters and times of events for testing for the demographic scenarios described in Table 2 and Fig 2. Between the brackets [] are the host values obtained from anthropological references as shown in Table 2 of the main manuscript. Louse values were estimated using anthropological references under different louse infestation levels as well as different generational times. S2 Table. Population size priors considering 10% and 50% louse infestation level. S3 Table. Louse time priors are expressed considering number of generations of lice back in time assuming a generation time of 27 and 36 days, respectively. The values for hominins refer to years. S4 Table. Genetic differentiation. Louse pairwise genetic differentiation based on FST values from 15 microsatellite loci among sites where 8 or more lice were collected. Sample sizes are in parentheses following population name. Values below the diagonal indicate FST values, p-values are shown above the diagonal; * indicates significant at nominal alpha level of 0.05. (ZIP) [file pone.0293409.s002.zip › PONE-D-22-28588_R1_S2_S3_Tables.pdf]

S2 and S3 Tables. Prior distributions of population sizes parameters and times of events for testing the demographic models described in Table 2 and Fig 2. Between the brackets [] are the host values obtained from anthropological references as shown in Table 2 of the main manuscript. Louse values were estimated using anthropological references under different louse infestation levels as well as different generational times.

S2 Table. Population size priors considering 10% and 50% louse infestation level.

Historic Events		10% Parasitism	50% Parasitism
Effective population	N	Minimum-Maximum	Minimum-Maximum
Louse Ancestral Population	N_A	300-6,000	7,500-150,000
[Ancestral hominins]		[300-6,000]	
Louse cluster I	N_1	300-6,000	7,500-150,000
[AMH]		[300-6,000]	
Louse cluster II	N_2	300-6,000	7,500-150,000
[Neanderthals]		[300-6,000]	
Louse Hybrids	N_3	45-900	1,125-22,500
[Hybrids between AMH and Neanderthals]		[?]	

S3 Table. Louse time priors are expressed considering number of generations of lice back in time assuming a generation time of 27 and 36 days, respectively. The values for hominins refer to years.

Historic Events		27 days	36 days
Time of Events	Time	Minimum-Maximum	Minimum-Maximum
Time of split clusters I and II	t_S	7,800,000 - 10,400,000	6,000,000 - 8,000,000
[Split between AMH and Neanderthals]		[600,000-800,000y]	
T1 ancestral polymorphism from cII	T1	1,560,000 - 1,820,000	1,200,000 - 1,400,000
[Split between AMH and Neanderthals		[120,000 - 140,000y]	
T2 ancestral polymorphism from cI	T2	650,000 - 1,040,000	500,000 - 800,000
[Presence of AMH in Middle East]		[50,000-80,000y]	
Time of ancestral admixture	t_{aA}	650,000 - 1,040,000	500,000 - 800,000
[AMH-Neanderthals in sympatry]		[50,000-80,000y]	
Times of Recent Admixture			
Time of admixture E1	T_{aE1}	1,300 - 13,000	1,000 - 10,000
[European colonization		[100 – 1,000y]	
Time of admixture E2	T_{aE2}	520 - 1,300	400 - 1000
[Migration after World Wars]		[40 – 100y]	
Time of admixture E1	T_{aG}	130 - 520	100 - 400
[Initial Globalization]		[10 – 40y]	
Time of admixture E1	T_{aC}	13 - 130	10 - 100
[Current time -t sampling]		[1 – 10y]	
