# Supplementary material for: Nuclear genetic diversity of head lice sheds light on human dispersal around the world
Source: PLoS One. 2023 Nov 8;18(11):e0293409. doi: 10.1371/journal.pone.0293409 (PMC10631634; doi:10.1371/journal.pone.0293409)
Supplement: S2 File — Groups reflect their geographic distribution and STRUCTURE clustering: Africa-Israel-Spain (AIS), South and Southeast Asia (SAs), East Asia (EAs), Europe (E), North America (NA), Mesoamerica (MA), Central America (CA), and South America (SAm). S2_S3 Tables. Prior distributions of population sizes parameters and times of events for testing for the demographic scenarios described in Table 2 and Fig 2. Between the brackets [] are the host values obtained from anthropological references as shown in Table 2 of the main manuscript. Louse values were estimated using anthropological references under different louse infestation levels as well as different generational times. S2 Table. Population size priors considering 10% and 50% louse infestation level. S3 Table. Louse time priors are expressed considering number of generations of lice back in time assuming a generation time of 27 and 36 days, respectively. The values for hominins refer to years. S4 Table. Genetic differentiation. Louse pairwise genetic differentiation based on FST values from 15 microsatellite loci among sites where 8 or more lice were collected. Sample sizes are in parentheses following population name. Values below the diagonal indicate FST values, p-values are shown above the diagonal; * indicates significant at nominal alpha level of 0.05. (ZIP) [file pone.0293409.s002.zip › S1 Table (1).pdf]

S1 Table. Detail of the human louse samples used in the current study. Groups reflect their geographic region: Africa-Israel-Spain (AIS), South and Southeast Asia (SAs), East Asia (EAs), Europe (E), North America (NA), Mesoamerica (MA), Central America (CA), and South America (SAm).

Geographic region	Group	Country	Site Code	No. of samples	City, State or Province	Collectors
Africa	AIS	Rwanda	Rw	1	Unknown	D. Raoult
West Asia	AIS	Israel	Isr	2	Unknown	K. Shepherd
Central Asia	SAs	Nepal	Np	1	Kathmandu	K. Yoshizawa
South and Southeast Asia	SAs	Thailand	Th	1	Sanklaburi, Kanchanaburi	K. Shepherd
		Cambodia	Cam	22	Batambang, Batambang	K. Shepherd
		Laos	Ls	4	Unknown	J-P. Hugot
East Asia	EAs	Mongolia	Mn	10	Unknown	N/A
Europe	E	Turkey	Tur	12	Sivas	S. Degerli
	E	Croatia	Croa	6	Unknown	M. Braks
	E	Norway	Nw	1	Tromso	K. Gravningen
	E	Netherlands	Neth	64	Unknown	M. Braks
	E	France	Fr	3	Montpellier	F. Delsuc
	E	United Kingdom	UK	27	Unknown	N. Hill
	AIS	Spain	Spain	3	Unknown	K. Shepherd
North America	NA	USA	Oce	25	New York city area	C. Gilbert
	NA	USA	SF	1	San Francisco, California	M. Mitchell
	NA	USA	Martin	1	Martin County, Florida	K. Shepherd
	NA	USA	WPB	1	West Palm Beach, Florida	K. Shepherd
	NA	USA	Nash	18	Nashville, Tennessee	A. Irwin
	NA	USA	MC	22	Missouri City, Texas	P. Warner
Meso America	MA	Mexico	MX-1	18	Tetitlán, Guerrero	A. González-Oliver
		Mexico	DF	1	México D.F.	
Central America	CA	Honduras	Hon	20	San Francisco, Zamorano Valley	K. Shepherd
South America	SAm	Peru	Peru	1	Unknown	J. Ungvari-Martin
		Argentina	Arg	9	Buenos Aires	A. Toloza
Total		19	25	274		
