# Supplementary material for: Nuclear genetic diversity of head lice sheds light on human dispersal around the world
Source: PLoS One. 2023 Nov 8;18(11):e0293409. doi: 10.1371/journal.pone.0293409 (PMC10631634; doi:10.1371/journal.pone.0293409)
Supplement: S2 File — Groups reflect their geographic distribution and STRUCTURE clustering: Africa-Israel-Spain (AIS), South and Southeast Asia (SAs), East Asia (EAs), Europe (E), North America (NA), Mesoamerica (MA), Central America (CA), and South America (SAm). S2_S3 Tables. Prior distributions of population sizes parameters and times of events for testing for the demographic scenarios described in Table 2 and Fig 2. Between the brackets [] are the host values obtained from anthropological references as shown in Table 2 of the main manuscript. Louse values were estimated using anthropological references under different louse infestation levels as well as different generational times. S2 Table. Population size priors considering 10% and 50% louse infestation level. S3 Table. Louse time priors are expressed considering number of generations of lice back in time assuming a generation time of 27 and 36 days, respectively. The values for hominins refer to years. S4 Table. Genetic differentiation. Louse pairwise genetic differentiation based on FST values from 15 microsatellite loci among sites where 8 or more lice were collected. Sample sizes are in parentheses following population name. Values below the diagonal indicate FST values, p-values are shown above the diagonal; * indicates significant at nominal alpha level of 0.05. (ZIP) [file pone.0293409.s002.zip › S4 Table.pdf]

S4 Table. Genetic differentiation. Louse pairwise genetic differentiation based on F_{ST} values from 15 microsatellite loci among sites where 8 or more lice were collected. Sample sizes are in parentheses following population name. Values below the diagonal indicate F_{ST} values, *p*-values are shown above the diagonal; * indicates significant at nominal alpha level of 0.05

	Tur	Neth	UK	Cam	Mn	Oce	Nash	MC	MEX	Hon
Tur (12)		*	*	*	*	*	*	*	*	*
Neth (64)	0.350		*	*	*	*	*	*	*	*
UK (27)	0.136	0.309		*	*	*	*	*	*	*
Cam (22)	0.335	0.601	0.421		*	*	*	*	*	*
Mn (10)	0.355	**0.663**	0.465	0.183		*	*	*	*	*
Oce (25)	0.169	0.267	0.109	0.437	0.484		*	*	*	*
Nash (18)	0.182	0.372	0.163	0.435	0.485	***0.053***		*	*	*
MC (22)	0.107	0.357	0.148	0.329	0.355	0.109	0.102		*	*
MX (19)	0.158	0.361	0.101	0.363	0.427	0.188	0.227	0.157		*
Hon (20)	0.338	0.596	0.374	0.227	0.316	0.400	0.403	0.260	0.318	
Arg (9)	0.223	0.513	0.229	0.275	0.313	0.236	0.267	0.106	0.185	0.183
