# Supplementary figures and images for: Nuclear genetic diversity of head lice sheds light on human dispersal around the world
Source: PLoS One. 2023 Nov 8;18(11):e0293409. doi: 10.1371/journal.pone.0293409 (PMC10631634; doi:10.1371/journal.pone.0293409)

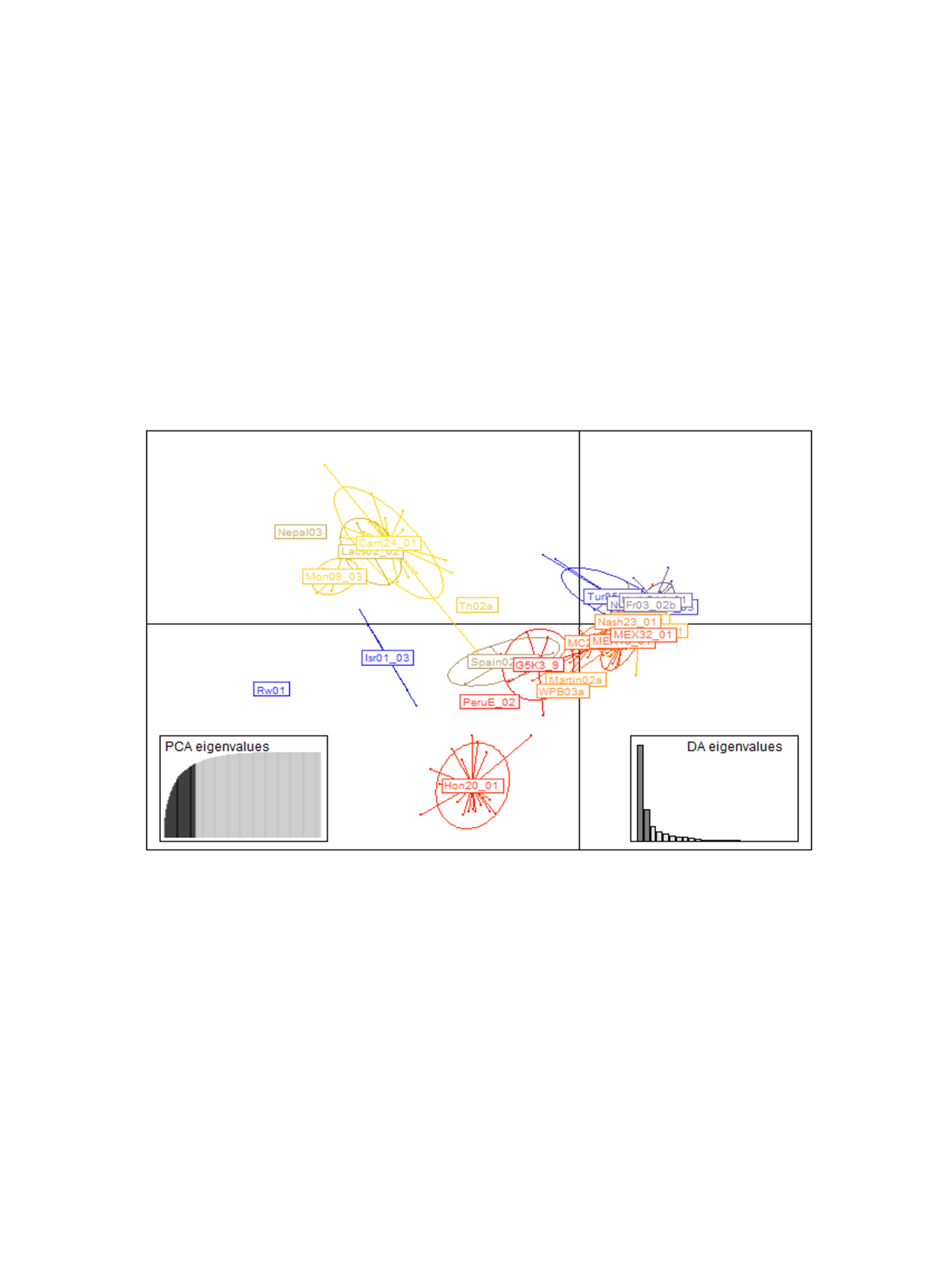

Supplement: S1 Fig — Cluster membership of each louse is depicted by a different colour inside their 95% inertia ellipses. DA eigenvalues are shown in the inserted graphs at the bottom right, whereby the numbers of the discriminants plotted against each other are indicated by dark grey and the remaining discriminants retained for the analysis in light grey. A total of 10 axes were retained in this DAPC, this is referred to the n-dim function in the adegenet package, and indicates the number of retained DAPC axes, which is affected by both the number of PCA axes and DA axes retained. (TIF) [file pone.0293409.s003.tif]

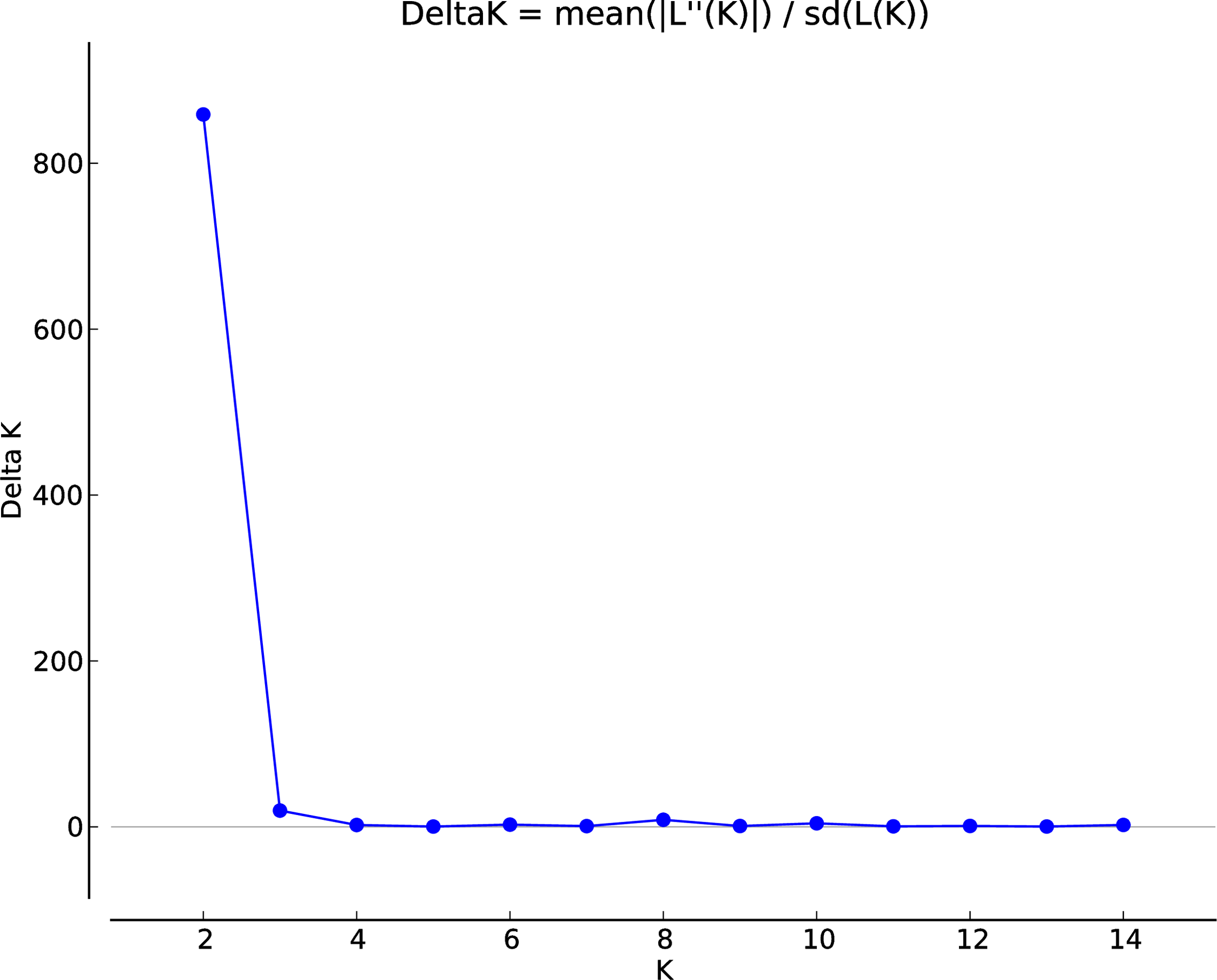

Supplement: S2 Fig — ΔK statistic of Evanno et al. [39] from STRUCTURE analysis from Fig 3. K = 2 (ΔK = 858.853). (TIF) [file pone.0293409.s004.tif]

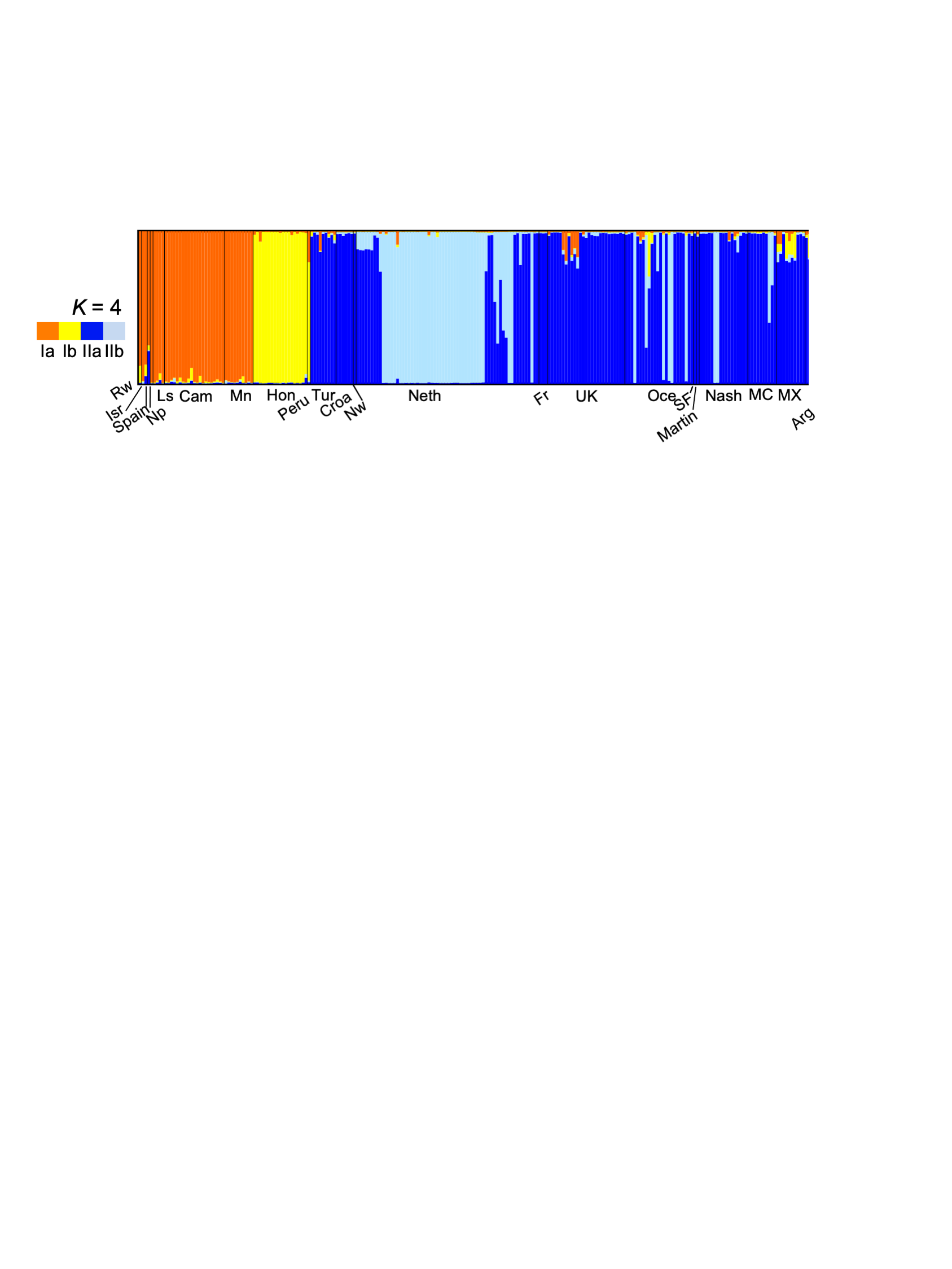

Supplement: S3 Fig — An ancestry threshold value of q-values as suggested in [38] was used because it is efficient and accurate at differentiating between non-interbreed and hybrids. Only individuals with q-value between 0 and < 0.20 or > 0.8 and 1 are classified as non-admixed and were included in this analysis. (TIF) [file pone.0293409.s005.tif]

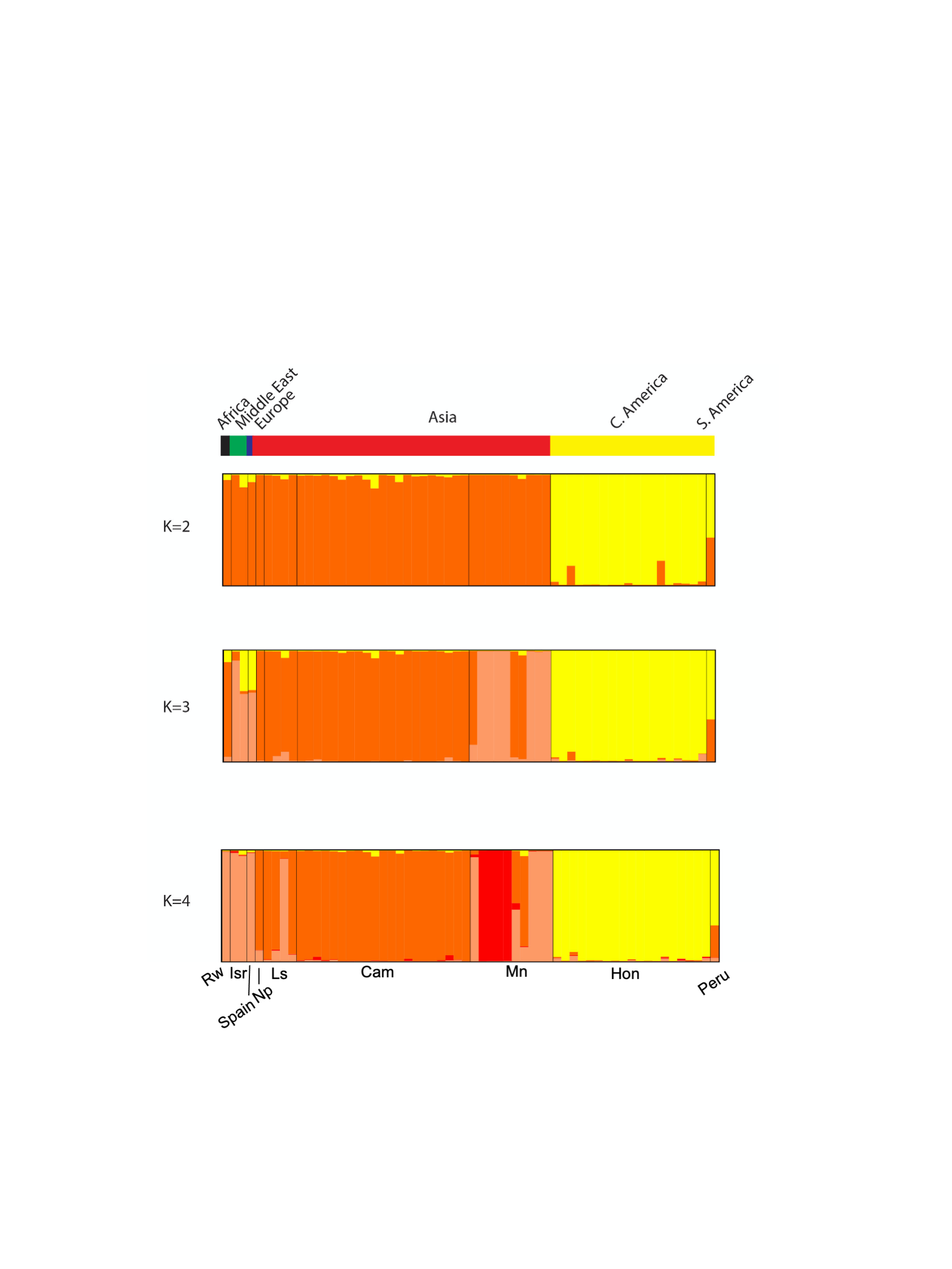

Supplement: S4 Fig — STRUCTURE plot including only non-admixed lice (no hybrids). (TIF) [file pone.0293409.s006.tif]

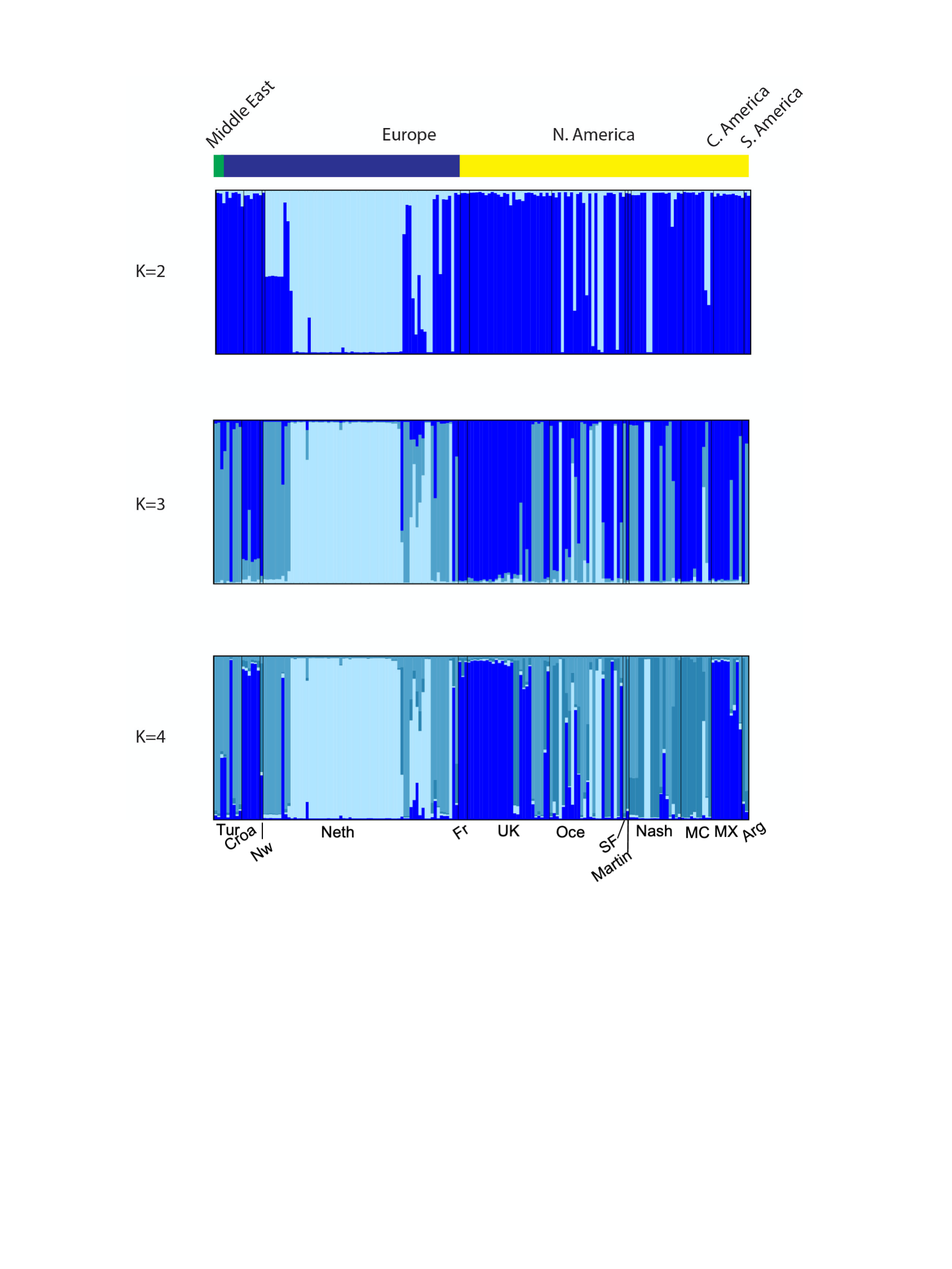

Supplement: S5 Fig — STRUCTURE plot including only non-admixed lice (no hybrids). (TIF) [file pone.0293409.s007.tif]

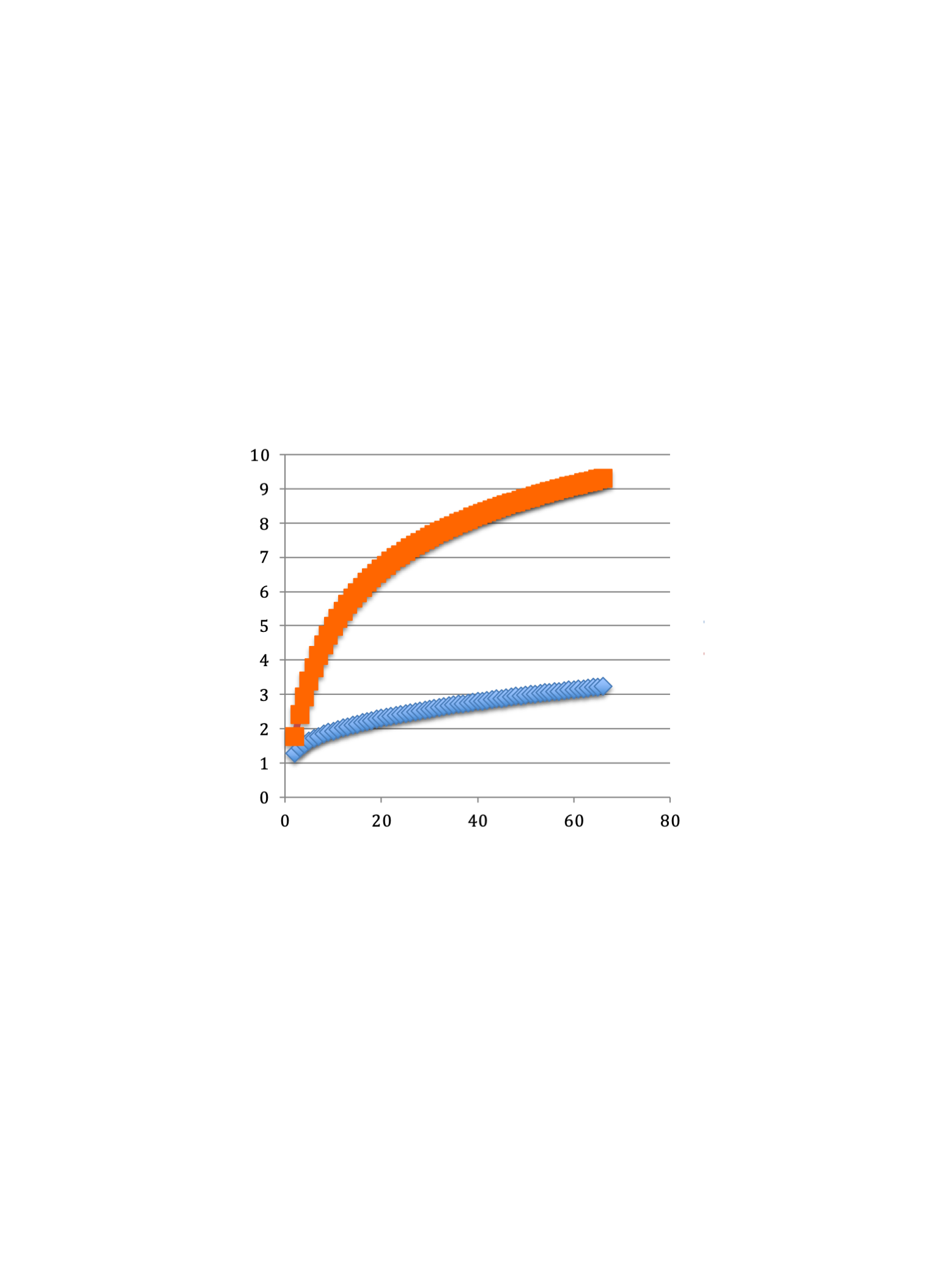

Supplement: S6 Fig — Allelic richness per nuclear genetic cluster (cI, orange and cII, blue) using the rarefaction method implemented in the computer program ADZE (Allelic Diversity Analyzer) Version 1.0 [44]. Because allelic richness measurements considered sample size, the rarefaction method allows the estimation of allelic richness for different random subsamples of size “g” from the populations [45, 46]. (TIF) [file pone.0293409.s008.tif]

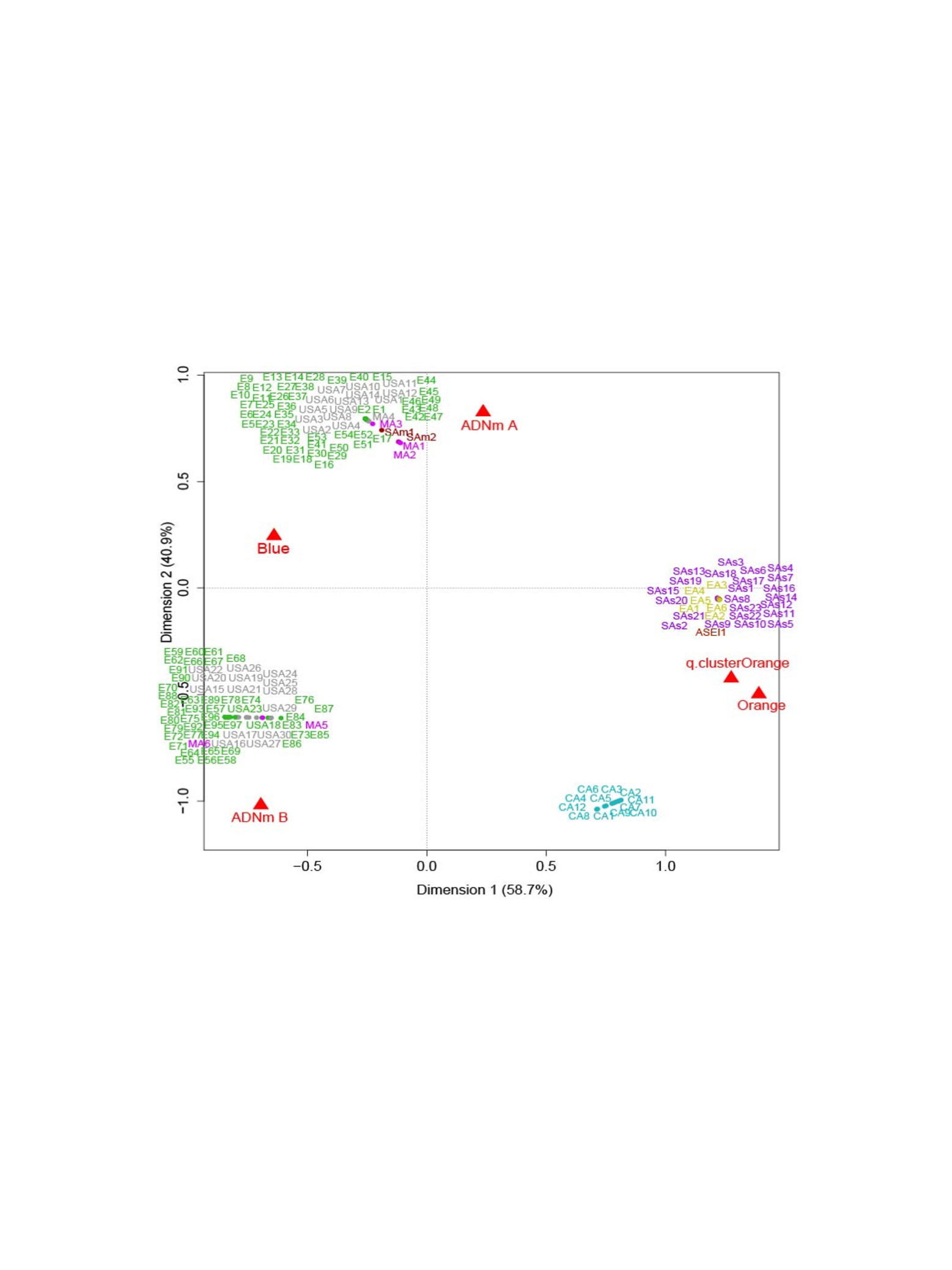

Supplement: S7 Fig — CA of the nuclear cluster membership frequency and the mitochondrial haplogroup of head lice. (TIF) [file pone.0293409.s009.tif]

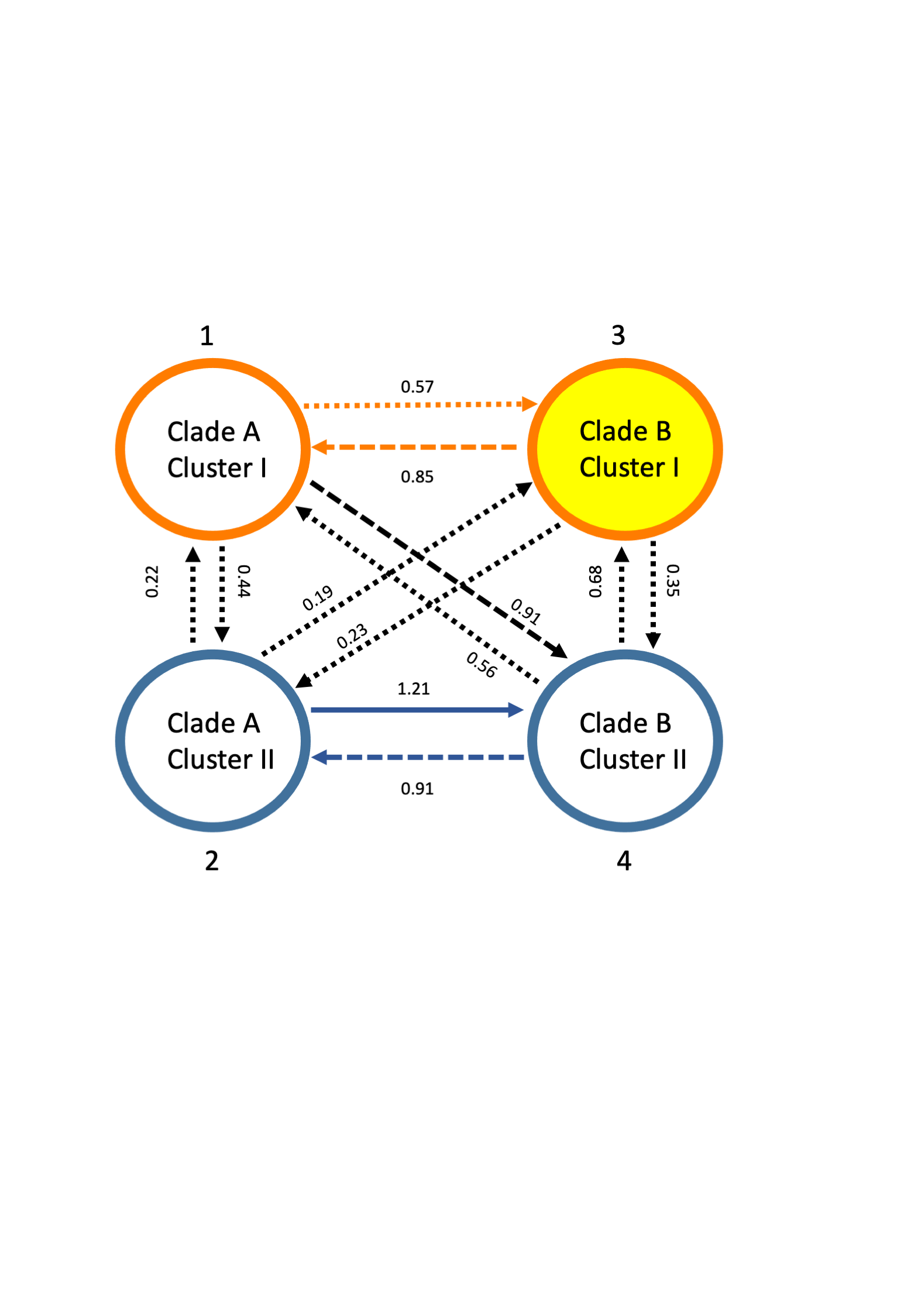

Supplement: S8 Fig — Full migration model with four units defined by both the nuclear clusters inferred from STRUCTURE and the mitochondrial haplogroups: unit 1) cI–mtDNA A; unit 2) cII–mtDNA A, unit 3) cI–mtDNA B; and unit 4) and cII–mtDNA B. Values above and below the arrows indicate migration rates, arrows indicated directionality. (TIF) [file pone.0293409.s010.tif]

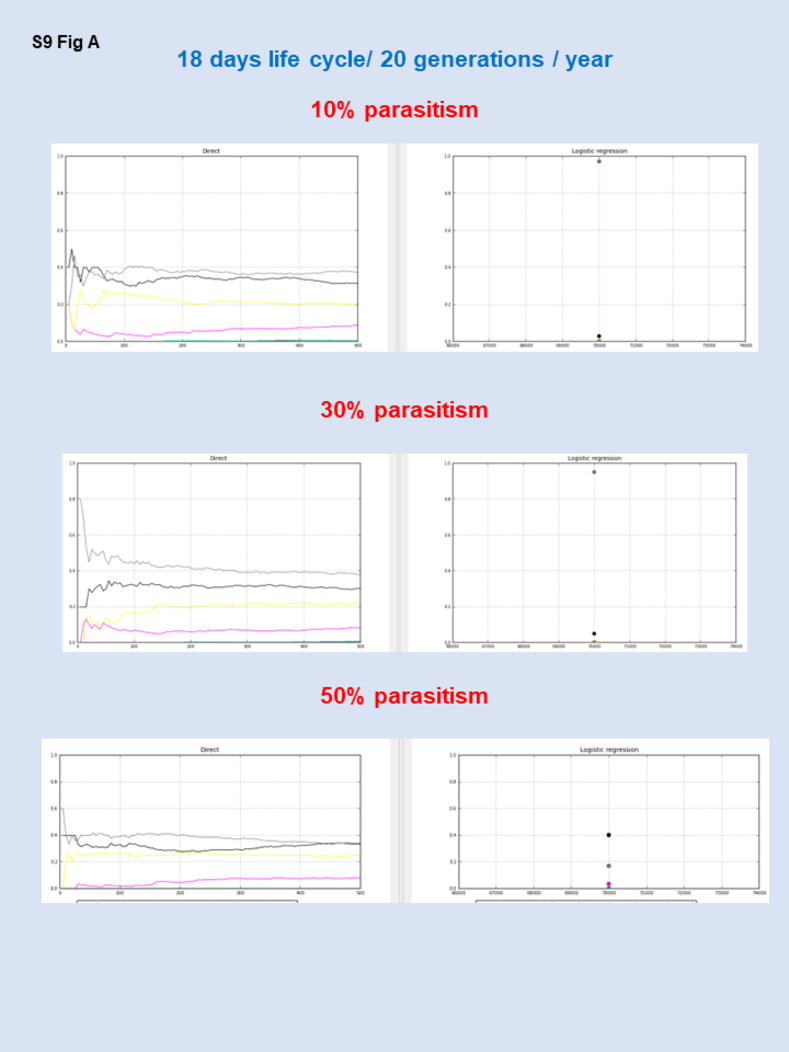

Supplement: S9 Fig — Louse infestation included three levels: 10% (S9 Fig A), 30% (S9 Fig B) and 50% (S9 Fig C), where 10% indicates that 10% of the hominin population have 10 lice per head, the 30% level considers that each head would have 20 lice and only 30% of the population is infested, while the 50% reflects high level of parasitism with 50 lice per head in 50% of the population. For this study we considered the time of head lice maturation from egg to reproductive male adult and egg-laying-adult female of 18 days, 27 days and 36 days. Each demographic model was color-coded as follow Scenario 1 –green, Scenario 2 –red, Scenario 3 –light blue, Scenario 4 –pink, Scenario 5 –yellow, Scenario 6 –black, Scenario 7 –grey. (ZIP) [file pone.0293409.s011.zip › PONE-D-22-28588_R1_S9 Fig A.tif]

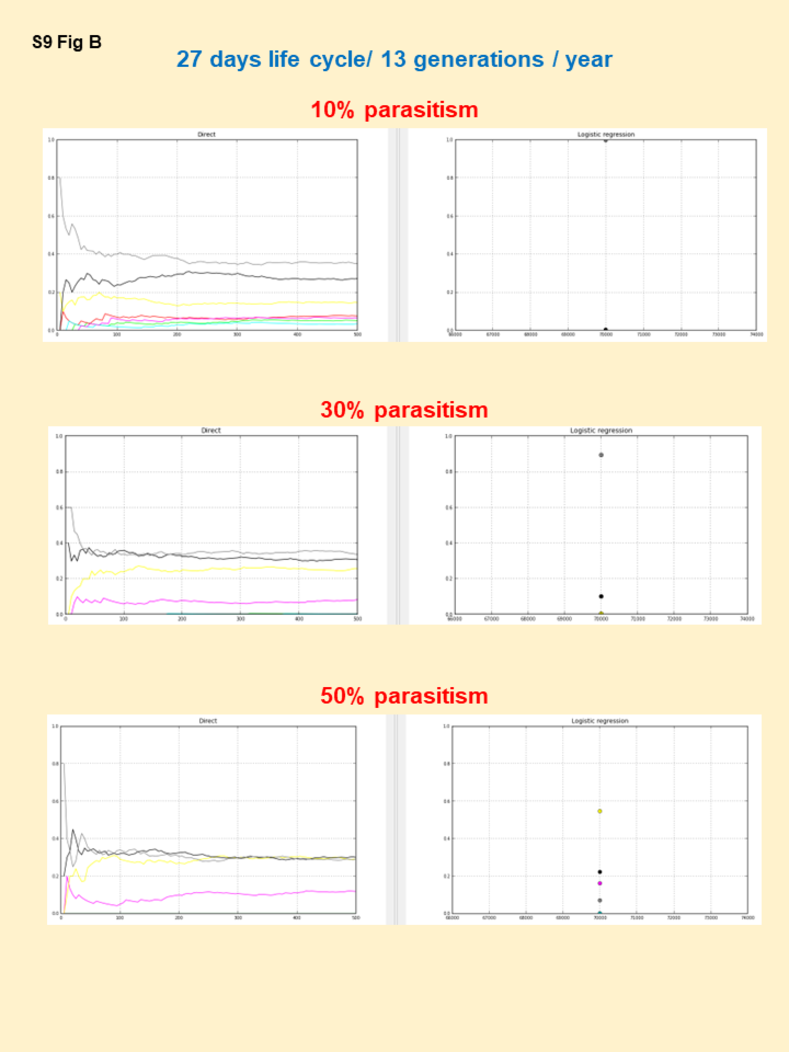

Supplement: S9 Fig — Louse infestation included three levels: 10% (S9 Fig A), 30% (S9 Fig B) and 50% (S9 Fig C), where 10% indicates that 10% of the hominin population have 10 lice per head, the 30% level considers that each head would have 20 lice and only 30% of the population is infested, while the 50% reflects high level of parasitism with 50 lice per head in 50% of the population. For this study we considered the time of head lice maturation from egg to reproductive male adult and egg-laying-adult female of 18 days, 27 days and 36 days. Each demographic model was color-coded as follow Scenario 1 –green, Scenario 2 –red, Scenario 3 –light blue, Scenario 4 –pink, Scenario 5 –yellow, Scenario 6 –black, Scenario 7 –grey. (ZIP) [file pone.0293409.s011.zip › PONE-D-22-28588_R1_S9 Fig B.tif]

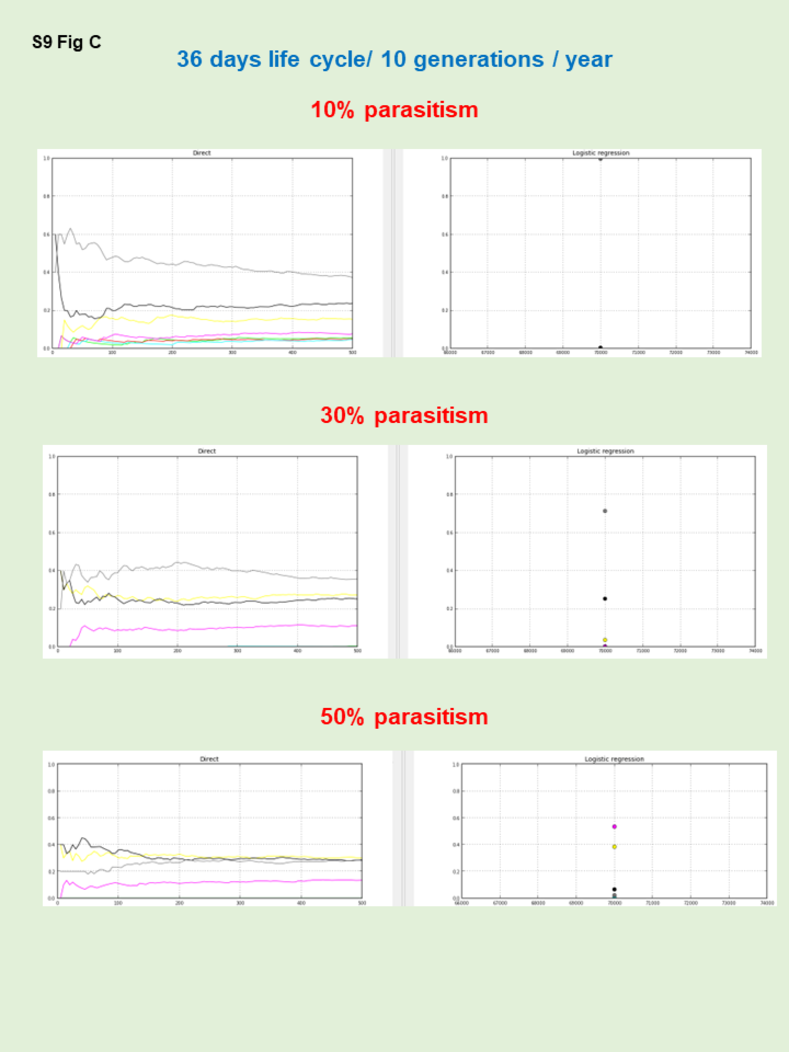

Supplement: S9 Fig — Louse infestation included three levels: 10% (S9 Fig A), 30% (S9 Fig B) and 50% (S9 Fig C), where 10% indicates that 10% of the hominin population have 10 lice per head, the 30% level considers that each head would have 20 lice and only 30% of the population is infested, while the 50% reflects high level of parasitism with 50 lice per head in 50% of the population. For this study we considered the time of head lice maturation from egg to reproductive male adult and egg-laying-adult female of 18 days, 27 days and 36 days. Each demographic model was color-coded as follow Scenario 1 –green, Scenario 2 –red, Scenario 3 –light blue, Scenario 4 –pink, Scenario 5 –yellow, Scenario 6 –black, Scenario 7 –grey. (ZIP) [file pone.0293409.s011.zip › PONE-D-22-28588_R1_S9 Fig C.tif]
